# Age‐associated differences in the breast cancer immune microenvironment

**DOI:** 10.1002/2056-4538.70115

**Published:** 2026-11-03

**Authors:** Rasmus OC Humlevik, Ying Chen, Tor A Klingen, Anna KM Sæle, Amalie A Svanøe, Ulrikke Hugaas, Amalie B Kvamme, Amalie F Tegnander, Ingeborg Winge, Turid Aas, Anette Heie, Lars A Akslen, Elisabeth Wik, Erling A Hoivik

**Affiliations:** ^1^ Centre for Cancer Biomarkers CCBIO, Department of Clinical Medicine University of Bergen Bergen Norway; ^2^ Department of Pathology Haukeland University Hospital Bergen Norway; ^3^ Fürst Medical Laboratorium Oslo Norway; ^4^ Faculty of Health Sciences Oslo Metropolitan University Oslo Norway; ^5^ Department of Pathology Vestfold Hospital Tønsberg Norway; ^6^ Department of Surgery Haukeland University Hospital Bergen Bergen Norway

**Keywords:** breast cancer, young age, immune cells, TILs, TAMs, PD‐L1, FOXP3, CD163, Tregs, tumor immune microenvironment

## Abstract

Young age is associated with poorer outcomes and more aggressive clinicopathological features in breast cancer, but the biological underpinnings are incompletely understood. With the emergence of infiltrating immune cell tissue‐based biomarkers, we aimed to investigate a relation to age by mapping of CD45, CD3, CD8, FOXP3, and CD163 positive cells. We examined a population‐based cohort of women under the age of 50 years (BCY‐B cohort, *n =* 311) in comparison to a previously reported series of patients aged 50–69 (TBC cohort, *n =* 282) years. The BCY‐B cohort was additionally stained for two PD‐L1 markers. Young age (under 50 years) was not associated with higher levels of tumor‐infiltrating lymphocytes (TILs), represented by markers such as CD45 and CD3. However, younger patients presented with lower levels of FOXP3‐positive TILs, both in luminal and non‐luminal subgroups, and higher levels of CD163‐positive tumor‐associated macrophages (TAMs) in combined analyses only. CD163 showed prognostic value when analyzing all patients together and within the luminal subgroups. For non‐luminal subgroups, CD3 expression was prognostic, while CD8 expression showed a trend. Notably, no age‐associated difference in prognostic value was observed for any marker. Among patients within the BCY‐B cohort (aged under 50 years), those below 40 years demonstrated higher PD‐L1 tumor cell expression, also within the triple‐negative subgroup, when compared to those aged 40–49 years. In conclusion, our study found only limited differences in tumor‐infiltrating immune cells with age, with higher density of FOXP3‐positive TILs in patients aged 50 years or above. Within the BCY‐B cohort (age under 50), patients aged under 40 years showed higher tumor expression of PD‐L1 when compared to those aged 40–49 years, suggesting age‐associated differences in immune checkpoint regulation.

## Introduction

The incidence of early onset breast cancer (BC, under 50 years of age) is on the rise in European countries and the United States [1, 2]. These patients present with a worse overall prognosis and more aggressive tumor characteristics compared to older BC patients. The tumors of younger patients are more often larger, of higher grade, with lymph node involvement, and hormone receptor negativity [3, 4, 5]. Young age is generally associated with a more efficient immune system, and young BC patients present with tumor characteristics that are linked with immune responses, such as high tumor cell proliferation and immune cell rich BC subtypes (triple‐negative and basal‐like) [6, 7]. Thus, the clinical utility of biomarkers and treatment effectiveness may be influenced by age [8].

There has been an increasing interest in elucidating the role of tumor‐infiltrating lymphocytes (TILs) and tumor‐associated macrophages (TAMs) in BC, driven by their involvement in the interface between the immune system and tumor cells [9]. TILs comprise various lymphocyte subsets, predominantly T cells, B cells, and natural killer (NK) cells, and are essential components of the tumor microenvironment [10]. TILs have been recognized as both prognostic and treatment predictive in non‐luminal, triple‐negative, and HER2‐positive BC [11, 12, 13]. However, the heterogeneity of immune cell subpopulations underscores the complexity of the immune system and immune responses in cancer. While certain immune cells exert anti‐tumor effects, others may facilitate tumor progression. Tumor‐associated macrophages exemplify this dual role as they can be polarized into either anti‐tumorigenic (M1‐like) or pro‐tumorigenic (M2‐like) phenotypes [14].

Some immune cell markers are applied as pan‐markers to reflect the overall lymphocyte and macrophage populations involved in tumor immunity. CD45, also known as leukocyte common antigen (LCA), serves as a general indicator of immune cell infiltration [15]. CD3 specifically identifies T lymphocytes, while CD8 marks cytotoxic T lymphocytes, playing a key role in anti‐tumor activity [16, 17]. More specific markers also exist. FOXP3 is a marker of regulatory T cells (Tregs), contributing to immunosuppression within the tumor microenvironment [18]. Similarly, CD163 is expressed by M2‐polarized macrophages, a subtype associated with tumor progression through immune suppression [19]. Together, these markers provide valuable insight into the composition and functional aspects of the immune microenvironment, potentially influencing tumor progression and patient outcomes.

Recent studies have identified additional immune components as critical for the immune function, in particular immune checkpoint indicators [20]. Immune checkpoints are molecules that modulate the initiation and intensity of immune responses [20]. In recent years, therapeutic targeting of immune checkpoints has been successful, particularly in triple‐negative breast cancer, where antibodies blocking the PD‐1/PD‐L1 interaction have demonstrated clinical benefit [21]. Thus, in groups of advanced BC, immunohistochemistry (IHC) assays are used to assess PD‐L1 expression and guide treatment decisions. The anti‐PD‐L1 antibody 22C3 clone is used to identify patients eligible for *pembrolizumab* therapy, while the SP142 antibody clone has been applied to predict response to *atezolizumab* [22, 23]. However, the US Food and Drug Administration (FDA) recently withdrew approval for *atezolizumab* in combination with *paclitaxel*, following the results from the IMpassion131 trial, which failed to show a survival benefit in metastatic triple‐negative BC [21].

Only a few studies have explored the relationship between age and infiltrating immune cells in breast cancer [24, 25, 26, 27, 28]. Overall, younger patients exhibit higher levels of immune cell markers. TILs have been found to be prognostic in young women with both hormone receptor positive/HER2 negative and triple negative tumors [29, 30]. However, current studies of BC are limited by small sample sizes and inconsistent results, highlighting the need for studies on larger, population‐based cohorts, focusing specifically on the age‐related differences in the tumor immune microenvironment. Understanding how age influences immune infiltration is critical for optimizing treatment strategies. This is particularly important given the increasing number of clinical trials incorporating immune biomarkers without accounting for age as a potential confounding factor. In this study, we examined tumor immune cell markers across age groups using population‐based cohorts of young and older BC patients.

## Materials and methods

### Ethics statement and patient consent

The study was approved by the Regional Committees for Medical and Health Research Ethics; Regional Ethics Committee of Western Norway (REK 2014/1984; BCY‐B cohort) and by the Regional Ethics Committee of Eastern Norway (REK #S‐08685d; TBC cohort). The study was performed in accordance with the Declaration of Helsinki (https://www.wma.net/policies-post/wma-declaration-of-helsinki/). Written informed consent was waived by the ethics committee. The national identification numbers of all patients were checked with the Registry of Withdrawal from Biological Research Consent by the Norwegian Institute of Public Health. None of the cases were listed in the registry.

### In‐house patient cohorts

In this study, we combined patient information and biospecimen materials from two Norwegian BC cohorts: Breast Cancer of the Young–Bergen (BCY‐B) cohort (*n =* 355; young BC cohort, 25–49 years) and Tønsberg Breast Cancer (TBC) cohort (*n =* 282; older BC cohort, 50–69 years) (supplementary material, Figure S1) [8, 31]. The BCY‐B cohort is a population‐based cohort comprising all women under 50 years, diagnosed with invasive breast cancer in Hordaland County from January 1996 to December 2003. Clinico‐pathologic variables and follow‐up data were available from a previous study from the BCY‐B research group [8]. Tumors with available tissue microarrays (TMAs) (*n =* 311) were further employed for immunohistochemical (IHC) assessment of the immune cell markers CD45, CD3, CD8, CD163, and FOXP3. The TBC cohort is a population‐based cohort of women aged 50 to 69 years who participated in the Norwegian Breast Cancer Screening Program (NBCSP) and were diagnosed with invasive breast cancer between February 2004 and December 2009 in Vestfold County. Clinico‐pathologic variables, follow‐up data, and IHC evaluations of the same immune cell markers were obtained from previously published work and used for comparative analyses after carefully assessing their compatibility [31].

### IHC staining

Three tissue cores (diameter 1.0 mm) were extracted from the invasive edge of the tumor and mounted in TMA recipient blocks, using a semi‐automated instrument (Minicore 3, Tissue Arrayer, Alphelys, France). From the TMA blocks, 4–5 μm thick sections were cut and mounted on poly‐lysine coated glass slides [8, 31]. Xylene and ethanol were used for deparaffination. A pressure cooker (35 s at 125 °C) was used for target retrieval for CD45 (pH 9) and CD163 (pH 6), while a microwave (20 min) was used for CD3, CD8 and FOXP3 (pH 9). Peroxidase block was used for all staining to prevent endogenous peroxidase activity. The primary antibodies were diluted using the following ratios and incubation time: CD45 (Agilent Dako, Glostrup, Denmark, M0701, mouse) 1:400 for 60 min; CD3 (Agilent Dako, A0452, rabbit) 1:200 for 30 min; CD8 (Agilent Dako, M7103, mouse) 1:100 for 30 min; FOXP3 (BD Pharmingen, Sandiego, CA, USA, 259D/C7, mouse) 1:20 for 30 min; CD163 (Leica, Newcastle upon Tyne, UK, NCL‐L‐CD163, mouse) 1:200 for 60 min. Secondary antibodies were incubated for 30 min. Finally, diaminobenzidine (DAB) as chromogen was added for 10 min and hematoxylin (S3301, Agilent Dako) as a counterstain for 10 min.

IHC staining for the PD‐L1 antibody 22C3 was performed with the DAKO platform and according to protocol from Agilent (Santa Clara, CA, USA; cat. no. GI10033). EnVision FLEX TRS, Low pH was used for target retrieval for 40 min at 97 °C. EnV FLEX Peroxidase‐Blocking Reagent (Agilent; cat. no. GV800) was used for endogenous enzyme block. The primary and secondary (EnV FLEX+ Mouse Linker) antibodies were incubated for 40 and 10 min. DAB chromogen was incubated for 5 min. Hematoxylin incubation for 3 min was used for counter stain.

IHC staining for the PD‐L1 antibody SP142 (Ventana Medical Systems, Inc., Tucson, AZ, USA) was performed with the Ventana Ultra (Ventana Medical Systems; cat. no. 05342716001) platform and according to protocol from Roche Diagnostics (Rotkreuz, Switzerland). Target retrieval was done with Cell Conditioner #1 for 24 min and OV PEROX IHBTR for 4 min for endogenous enzyme block. The primary antibody was incubated for 16 min. The OV HQ UNIV LINKR and OV HRP MULTIMER were both incubated for 8 min. DAB chromogen was incubated for 8 min. Hematoxylin incubation for 4 min was used for counter stain.

### IHC evaluation

For immune cell markers, all three tissue cores were evaluated at low magnification. The tissue core with the estimated highest number of positive immune cells was selected for exact counting. This core was then examined at high magnification, and the region with the highest density of positive immune cells (hotspots) was counted using an eyepiece graticule (10 × 10 grid lines, total 0.059 mm^2^) (Figures 1A, 3A). One grid area per patient was counted. To note, IHC evaluation on the TBC‐cohort used a different grid (10 × 10 grid lines, total of 0.096 mm^2^) [31]. The counting from both cohorts was then standardized by positive cells per mm^2^.

**Figure 1:**
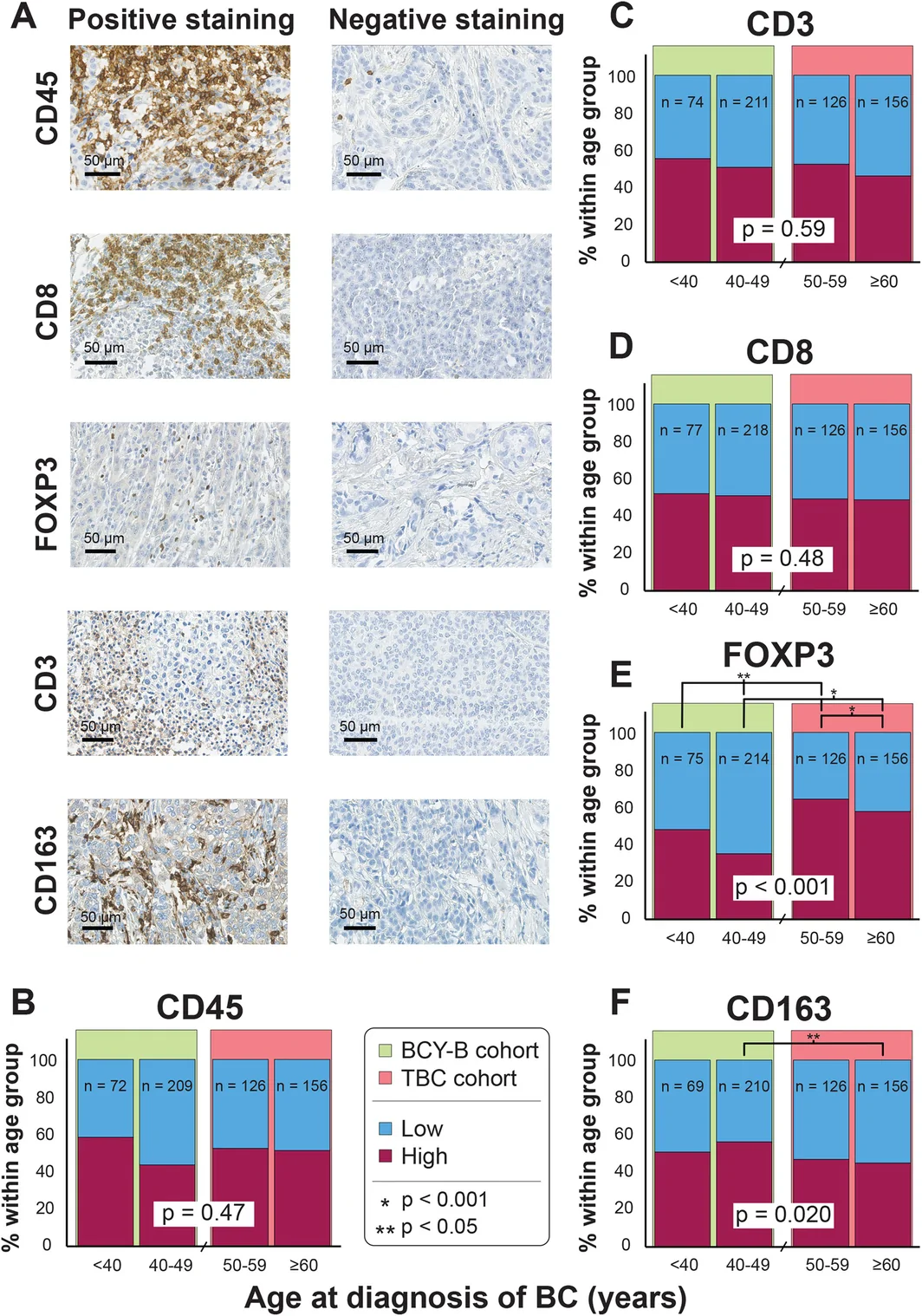
Immunohistochemical staining of immune markers and age‐related differences in tumor‐infiltrating leukocytes. (A) Immunohistochemical (IHC) evaluation of CD45, CD3, CD8, FOXP3, CD163 immune‐related markers demonstrating positive staining/high immune cell counts and sparse or negative staining/low immune cell counts. All images; scale bar, 50 μm. (B–E) Immunohistochemical (IHC) evaluation of immune markers across age groups; (B) CD45 (*n =* 563), (C) CD3 (*n =* 567), (D) CD8 (*n =* 577), (E) FOXP3 (*n =* 571), and (F) CD163 (*n =* 561). Data are combined from BCY‐B (young cohort, *n =* 311; light green) and TBC cohorts (older cohort, *n =* 282; pink). Markers are dichotomized by the median value. The *p* values are annotated by Pearson chi‐square comparing ages under versus over 50 years of age or indicated as * (*p* < 0.001) or ** (*p* < 0.05) in direct comparison between the specific age groups.

For the PD‐L1 markers, all three tissue cores were evaluated at high magnification. Staining was defined as positive or negative according to standard criteria and guidelines [32, 33], and location was noted (whether the staining was present in immune cells and/or tumor cells).

### Inter‐observer agreement and integration of cohorts

After evaluating the BCY‐B cohort, 70 cases of each immune cell staining from the TBC cohort were assessed to evaluate inter‐observer agreement (by ROCH). The Kappa (dichotomized by median) and ranked intraclass correlation (single measure, two‐way mixed) for each variable were, respectively: CD45, 0.63 and 0.89; CD3, 0.80 and 0.93; CD8, 0.66 and 0.86; FOXP3, 0.76 and 0.90; and CD163, 0.66 and 0.77, interpreted as strong and reliable.

To account for differences in graticule areas (0.059 and 0.096 mm^2^ eyepieces for the BCY‐B and TBC, respectively) and for inter observer variability of IHC‐scores, data from the two cohorts were standardized and adjusted. Cell counts for each marker were standardized to counts per mm^2^ in both datasets to obtain cell density as the metric (cells/mm^2^). To achieve data integration relative to the BCY‐B dataset, the standardized measurements from the TBC cohort (assessor #2 YC) were adjusted by the ratio of median values from the interobserver assessment to standardized measurements of the BCY‐B cohort (assessor #1 ROCH). The ratios for this calculation were as follows: CD45, 3.02; CD3, 2.41; CD8, 2.06; FOXP3, 2.44; and CD163, 0.57. The resulting harmonized data allowed for statistical assessment of the combined cohort. A sensitivity analysis was conducted on age‐related differences in immune cell infiltration before and after harmonization, demonstrating that the harmonization was a required step, and that the harmonization did not introduce any apparent changes in the key results either in the interobserver agreement cases or the full combined cohort (supplementary material, Figure S2, Table S1).

### Molecular subtype classification

The molecular classification was done according to the St. Gallen Guidelines 2013, with minor modifications [34]. Luminal A: ER‐ and/or PR‐positive, HER2‐negative and Ki67 low; Luminal B/HER2‐negative: ER‐ and/or PR‐positive, HER2‐negative and Ki67 high; Luminal B/HER2‐positive: ER‐ and/or PR‐positive, HER2‐positive; HER2‐positive (non‐luminal): ER‐ and PR‐negative and HER2‐positive; Triple‐negative: ER‐, PR‐, and HER2‐negative. Based on the study by Cheang and colleagues, who matched the Ki67 threshold to PAM50 subtype allocation, Ki67 was dichotomized based on the prespecified value corresponding to the 65th percentile of ER and/or PR‐positive HER2‐negative tumors, calculated as 8.7% in our data [8, 35]. Since this threshold was in the low range compared to other studies, a sensitivity analysis with thresholds at 10% and 14% was conducted, demonstrating that the threshold we applied did not affect our results with regard to survival and immune infiltration across subgroups (supplementary material, Figure S3, Table S2). Moreover, we mainly focused on the combined luminal versus non‐luminal subgroups, where this threshold did not influence the number of patients allocated to these groups.

### Statistical analyses

All statistical tests were analyzed using SPSS for Windows version 29 (IBM Corp, Armonk, NY, USA). Two‐sided *p* value ≤0.05 was considered significant. Associations between categorical variables were assessed using Pearson's chi‐squared test. Kaplan–Meier methods, with log‐rank test to compare different categories, were used for survival analyses. Entry date was time of diagnosis and breast cancer‐specific death was the outcome event. Patients who died from causes other than cancer were censored at the date of death. Cox's proportional hazards regression (Enter method) was used when testing for interactions and to calculate hazard ratios. The median value for each immune‐cell marker, calculated from the combined harmonized cohort, was used to dichotomize cases into low‐ and high‐level groups for comparisons across clinico‐pathologic variables and survival analyses. The 95% confidence intervals for the Kaplan–Meier curves were visualized using the survminer package in R (R Foundation for Statistical Computing, Vienna, Austria, version 4.6.2) and RStudio (Posit Software, PBC, Boston, MA, USA, version 2026.07.1).

## Results

### Increased aggressive clinico‐pathological features in young patients

Evaluation of clinico‐pathologic characteristics between the two cohorts revealed alignment with previously reported age‐related differences in BC patients [8]. Younger patients exhibited more aggressive tumor features, including ER/PR hormone receptor negativity, HER2 receptor positivity, lymph node involvement, larger tumor diameter, higher histologic grade, and elevated Ki‐67 expression, observed in younger age groups (Table 1, all *p* ≤ 0.016). Furthermore, younger age was significantly associated with non‐luminal, HER2^+^, and triple‐negative breast cancer.

**Table 1:** Clinico‐pathologic features of the BCY‐B (*n =* 311) and TBC cohorts (*n =* 282)

	BCY‐B (younger) cohort		TBC (older) cohort		*p*
	Under 40	40–49	50–59	60–69	
	*n* (%)	*n* (%)	*n* (%)	*n* (%)	
ER status					
Positive	36 (46.2)	175 (73.8)	104 (85.2)	141 (90.4)	<0.001
Negative	42 (53.8)	62 (26.2)	18 (14.8)	15 (9.6)	
PR status					
Positive	34 (43.6)	178 (75.1)	76 (62.3)	107 (68.6)	<0.001
Negative	44 (56.4)	59 (24.9)	46 (37.7)	49 (31.4)	
HER2 status					
Negative	51 (66.2)	209 (89.3)	110 (90.2)	141 (90.4)	<0.001
Positive	26 (33.8)	25 (10.7)	12 (9.8)	15 (9.6)	
Lymph node status					
Negative	33 (43.4)	131 (56.0)	82 (67.2)	103 (66.5)	0.001
Positive	43 (56.6)	103 (44.0)	40 (32.8)	52 (33.5)	
Tumor diameter					
≤20 mm	47 (60.3)	132 (56.7)	83 (68.0)	127 (81.4)	<0.001
>20 mm	31 (39.7)	101 (43.3)	39 (32.0)	29 (18.6)	
Tumor grade					
Grade 1	8 (11.0)	62 (27.3)	28 (23.0)	46 (29.5)	0.016
Grades 2 and 3	65 (89.0)	165 (72.7)	94 (77.0)	110 (70.5)	
Ki67					
Low	25 (33.3)	114 (49.1)	64 (52.5)	88 (56.4)	0.010
High	50 (66.7)	118 (50.9)	58 (47.5)	68 (43.6)	
Molecular subgroups					
Luminal A	16 (21.1)	101 (43.7)	62 (50.8)	85 (54.5)	<0.001
Luminal B/HER2 neg	16 (21.8)	75 (32.5)	36 (29.5)	47 (30.1)	
Luminal B/HER2 pos	10 (13.2)	15 (6.5)	7 (5.7)	11 (7.1)	
HER2, non‐luminal	16 (21.1)	10 (4.3)	5 (4.1)	4 (2.6)	
Triple negative	18 (23.7)	30 (13.0)	12 (9.8)	9 (5.8)	

The *p* values by Pearson's chi‐square test. Missing data: ER: *n =* 0; PR: *n =* 0; HER2: *n =* 4; lymph node status: *n =* 6; tumor diameter: *n =* 4; tumor grade: *n =* 15; Ki67: *n =* 8; molecular subtypes: *n* = 5.

### Higher levels of infiltrating immune cells are more strongly associated with aggressive clinico‐pathologic features in young patients

When assessing CD45, CD3, CD8, FOXP3, and CD163, we observed higher levels of immune cell markers in tumors with ER and PR negativity, high Ki67 and non‐luminal molecular subtypes, especially triple‐negative BC (supplementary material, Table S3, all *p* ≤ 0.027). Additionally, higher levels of CD3, CD8, FOXP3, and CD163 positive cells were associated with higher histologic grade (all *p* ≤ 0.011) and lymph node metastases, except FOXP3 (for all other markers, *p* ≤ 0.019).

When stratifying by age groups (<50 years versus ≥50 years), notable differences emerged in the relationship between the levels of immune cells and clinico‐pathologic features (Table 2). In patients under 50 years, higher levels of immune cells showed stronger associations with aggressive tumor characteristics than in the older patient group (50–69 years), particularly PR status [CD45: OR = 1.72 (1.05–2.82) versus 0.1.28 (0.77–2.10); CD8: OR = 1.82 (1.12–2.97) versus 1.40 (0.85–2.30); FOXP3: OR = 2.15 (1.30–3.55) versus 1.43 (0.85–2.40)], lymph node involvement [CD3: OR = 1.69 (1.05–2.70) versus 1.28 (0.78–2.12); CD8: OR = 2.00 (1.25–3.18) versus 1.13 (0.68–1.86)], and tumor grade [CD3: OR = 2.07 (1.17–3.68) versus 1.62 (0.94–2.77); CD8: OR = 1.95 (1.10–3.45) versus 1.13 (0.68–1.86); FOXP3: OR = 3.51 (1.73–7.09) versus 1.46 (0.85–2.50)]. Exceptions to this pattern were observed for CD163 and FOXP3 in relation to HER2 status and lymph node involvement, where the associations were stronger in the older patients [CD163: HER2 status: OR = 1.15 (0.61–2.20) versus 5.03 (1.96–12.89); FOXP3: HER2 status OR = 1.82 (0.97–3.41) versus 4.13 (1.39–12.31)].

**Table 2:** Clinico‐pathologic features and infiltrating immune cells across young and older age groups

		CD45 positive cell count			CD3 positive cell count			CD8 positive cell count			FOXP3 positive cell count			CD163 positive cell count		
		Low	High		Low	High		Low	High		Low	High		Low	High	
		*n* (%)	*n* (%)	*p*	*n* (%)	*n* (%)	*p*	*n* (%)	*n* (%)	*p*	*n* (%)	*n* (%)	*p*	*n* (%)	*n* (%)	*p*
ER status	Age <50 (years)															
	ER positive	103 (56.6)	79 (43.4)	0.015	104 (54.5)	87 (45.5)	0.002	106 (53.8)	91 (46.2)	0.015	134 (68.4)	62 (31.6)	<0.001	99 (52.1)	91 (47.9)	<0.001
	ER negative	41 (41.4)	58 (58.6)		33 (35.1)	61 (64.9)		38 (38.8)	60 (61.2)		44 (47.3)	49 (52.7)		27 (30.3)	62 (69.7)	
	Age ≥50 (years)															
	ER positive	128 (51.4)	121 (48.6)	0.003	136 (54.6)	113 (45.4)	0.001	141 (56.6)	108 (43.4)	<0.001	108 (43.4)	141 (56.6)	<0.001	151 (60.6)	98 (39.4)	<0.001
	ER negative	8 (24.2)	25 (75.8)		8 (24.2)	25 (75.8)		5 (15.2)	28 (84.8)		3 (9.1)	30 (90.9)		6 (18.2)	27 (81.8)	
PR status	Age <50 (years)															
	PR positive	103 (56.3)	80 (43.7)	0.021	101 (52.9)	90 (47.1)	0.021	105 (53.6)	91 (46.4)	0.021	133 (67.9)	63 (32.1)	0.001	96 (50.3)	95 (49.7)	0.012
	PR negative	41 (41.8)	57 (58.2)		36 (38.3)	58 (61.7)		39 (39.4)	60 (60.6)		45 (48.4)	48 (51.6)		30 (34.1)	58 (65.9)	
	Age ≥50 (years)															
	PR positive	93 (50.0)	93 (50.0)	0.41	104 (55.9)	82 (44.1)	0.023	102 (54.8)	84 (45.2)	0.15	78 (41.9)	108 (58.1)	0.22	115 (61.8)	71 (38.2)	0.004
	PR negative	43 (44.8)	53 (55.2)		40 (41.7)	56 (58.3)		44 (45.8)	52 (54.2)		33 (34.4)	63 (65.6)		41 (43.7)	54 (57.3)	
HER2 status	Age <50 (years)															
	HER2 negative	118 (51.5)	111 (48.5)	0.84	113 (48.1)	122 (51.9)	0.67	117 (48.3)	125 (51.7)	0.93	153 (63.7)	87 (36.3)	0.057	105 (45.3)	127 (54.7)	0.71
	HER2 positive	25 (50.0)	25 (50.0)		21 (44.7)	26 (55.3)		25 (49.0)	26 (51.0)		23 (48.9)	24 (51.1)		19 (42.2)	26 (57.8)	
	Age ≥50 (years)															
	HER2 negative	126 (49.4)	129 (50.6)	0.22	132 (51.8)	123 (48.2)	0.45	136 (53.3)	119 (46.7)	0.11	107 (42.0)	148 (58.0)	0.006	151 (59.2)	104 (40.8)	<0.001
	HER2 positive	10 (37.0)	17 (63.0)		12 (44.4)	15 (55.6)		10 (37.0)	17 (63.0)		4 (14.8)	23 (85.2)		6 (22.2)	21 (77.8)	
N status	Age <50 (years)															
	Negative	81 (56.3)	63 (43.8)	0.073	80 (54.1)	68 (45.9)	0.020	86 (57.7)	63 (42.3)	0.001	99 (64.7)	54 (35.3)	0.25	71 (48.0)	77 (52.0)	0.27
	Positive	60 (45.5)	72 (54.5)		53 (40.2)	79 (59.8)		55 (39.0)	86 (61.0)		76 (58.0)	55 (42.0)		52 (41.3)	74 (58.7)	
	Age ≥50 (years)															
	Negative	91 (48.7)	96 (51.3)	0.90	99 (52.9)	88 (47.1)	0.42	98 (51.9)	90 (48.1)	0.83	76 (40.6)	111 (59.4)	0.58	113 (60.4)	74 (39.6)	0.030
	Positive	45 (47.9)	49 (52.1)		45 (47.9)	49 (52.1)		48 (51.1)	46 (48.9)		35 (37.2)	59 (62.8)		44 (46.8)	50 (53.2)	
Tumor Size	Age <50 (years)															
	≤20 mm	82 (51.6)	77 (48.4)	0.98	78 (48.4)	83 (51.6)	0.98	83 (49.7)	84 (50.3)	0.62	107 (66.0)	55 (34.0)	0.087	81 (50.9)	78 (49.1)	0.046
	>20 mm	61 (51.7)	57 (48.4)		58 (48.3)	62 (51.7)		58 (46.8)	66 (53.2)		69 (56.1)	54 (43.9)		45 (38.8)	71 (61.2)	
	Age ≥50 (years)															
	≤20 mm	103 (48.1)	111 (51.9)	0.95	106 (49.5)	108 (50.5)	0.36	106 (49.5)	108 (50.5)	0.18	89 (41.6)	125 (58.4)	0.17	123 (57.5)	91 (42.5)	0.28
	>20 mm	33 (48.5)	35 (51.5)		38 (55.9)	30 (44.1)		40 (58.8)	28 (41.2)		22 (32.4)	46 (67.6)		34 (50.0)	34 (50.0)	
Tumor Grade	Age <50 (years)															
	Low (grade 1 and 2)	32 (57.1)	24 (42.9)	0.31	39 (62.9)	23 (37.1)	0.009	39 (63.9)	22 (36.1)	0.009	50 (83.3)	10 (16.7)	<0.001	35 (58.3)	25 (41.7)	0.023
	High (grade 3)	104 (49.5)	106 (50.5)		92 (44.0)	117 (56.0)		99 (45.0)	121 (55.0)		122 (57.0)	92 (43.0)		85 (41.7)	119 (58.3)	
	Age ≥50 (years)															
	Low (grades 1 and 2)	40 (52.6)	36 (47.4)	0.37	45 (59.2)	31 (40.8)	0.096	43 (56.6)	33 (43.4)	0.33	35 (46.1)	41 (53.9)	0.16	55 (72.4)	21 (27.6)	<0.001
	High (grade 3)	96 (46.6)	110 (53.4)		99 (48.1)	107 (51.9)		103 (50.0)	103 (50.0)		76 (36.9)	130 (63.1)		102 (49.5)	104 (50.5)	
KI67	Age <50 (years)															
	Low	71 (58.7)	50 (41.3)	0.034	71 (55.9)	56 (44.1)	0.017	69 (52.7)	62 (47.3)	0.25	105 (81.4)	24 (18.6)	<0.001	71 (57.3)	53 (42.7)	<0.001
	High	72 (45.9)	85 (54.1)		64 (41.6)	90 (58.4)		74 (46.0)	87 (54.0)		69 (44.2)	87 (55.8)		52 (34.7)	98 (65.3)	
	Age ≥50 (years)															
	Low	81 (52.6)	73 (47.4)	0.11	86 (55.8)	68 (44.2)	0.078	89 (57.8)	65 (42.2)	0.027	73 (47.4)	81 (52.6)	0.002	102 (66.2)	52 (33.8)	<0.001
	High	55 (43.0)	73 (57.0)		58 (45.3)	70 (54.7)		57 (44.5)	71 (55.5)		38 (29.7)	90 (70.3)		55 (43.0)	73 (57.0)	

Patients under 50 are from the BCY‐B cohort (*n =* 311), and patients over 50 years of age are from the TBC cohort (*n =* 282). The *p* values by Pearson's chi‐square test. Missing data: CD45: *n =* 30; CD3: *n =* 26; CD8: *n =* 16; FOXP3: *n =* 22; CD163: *n =* 32; ER: *n =* 0; PR: *n =* 0; HER2: *n =* 4; lymph node status: *n =* 6; tumor diameter: *n =* 4; tumor grade: *n =* 15; Ki67: *n =* 8.

### Levels of infiltrating immune cells across age groups

We next assessed immune cell levels across specific age groups using both a binary classification (<50 versus ≥50 years) and a more granular stratification (<40, 40–50, 50–59, ≥60 years). When comparing patients above and below 50 years, no significant differences were observed in levels of the immune cell markers CD45 and CD3 (Figure 1B,C). However, analyses of specific immune cell subsets revealed age‐related differences: FOXP3‐positive T cells were more abundant in patients ≥50 years, while CD163 positive TAMs were more prevalent in tumors of patients <50 years (Figure 1E,F; *p* < 0.001 and *p =* 0.020). No significant difference was observed for CD8 positive T cells (Figure 1D).

Further analyses using four age categories revealed additional differences. Patients <40 years presented a trend of higher levels of CD45‐ and FOXP3‐positive TILs compared to those aged 40–49 (Figure 1B, *p =* 0.060; Figure 1E, *p =* 0.051). In contrast, FOXP3 showed lower levels in patients <40 years compared to those aged 50–59 (Figure 1E, *p =* 0.021). Similarly, patients aged 40–49 presented significantly lower FOXP3 levels than both the 50–59 and ≥60 age groups (Figure 1E, both *p* < 0.001). For CD163, patients aged 40–49 demonstrated higher levels compared to those aged ≥60 (Figure 1F, *p =* 0.016). Stratifying by luminal versus non‐luminal molecular subtypes confirmed that the age‐related increase in FOXP3 infiltration persisted across both subgroups (Figure 2D, luminal *p* < 0.001; non‐luminal *p =* 0.009), whereas the difference in CD163 infiltration was not statistically significant (Figure 2E). The counts of CD8 positive cells showed a trend toward higher infiltration in older (over 50 years) non‐luminal patients (Figure 2C, *p =* 0.068), while CD45 and CD3 remained unchanged across subtypes and age groups (Figure 2A,B).

**Figure 2:**
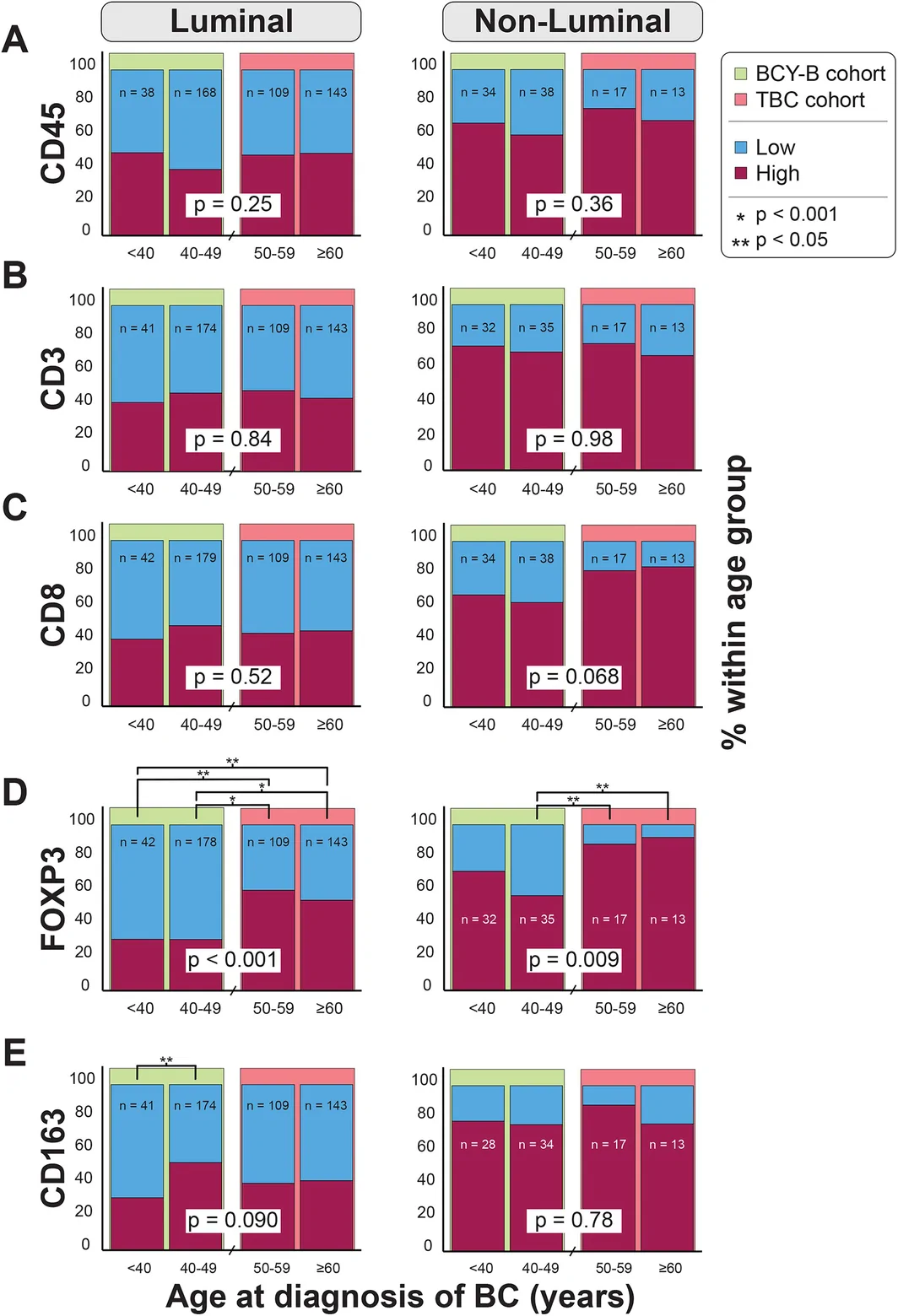
Age‐related differences in tumor‐infiltrating leukocytes across luminal and non‐luminal subgroups. Immunohistochemical evaluation of immune markers across age groups with stratification by luminal or non‐luminal subtypes; (A) CD45 (*n =* 560), (B) CD3 (*n =* 564), (C) CD8 (*n =* 575), (D) FOXP3 (*n =* 569), and (E) CD163 (*n =* 559). Luminal subgroup is defined as Luminal A, Luminal B HER2‐negative, and Luminal B HER2‐positive. Non‐Luminal subgroup is defined as HER2‐positive (non‐luminal) and triple‐negative. Data are from the two cohorts; BCY‐B (young cohort, *n =* 311; light green) and TBC cohort (older cohort, *n =* 282; pink). Markers are dichotomized by the median value. The *p* values are annotated by Pearson Chi‐Square comparing ages under versus over 50 years of age or indicated as * (*p* < 0.001) or ** (*p* < 0.05) in direct comparison between the specific age groups.

When investigating the specific age groups more closely, patients aged <40 and 40–49 with luminal tumors presented with significantly lower levels of FOXP3 positive cells compared to those aged 50–59 and ≥60 (Figure 2D, under 40: *p =* 0.001 and *p =* 0.010; 40–49 both *p* < 0.001). Among non‐luminal tumors, the same pattern was observed only in the 40–49 age group (Figure 2D, *p =* 0.025 and *p =* 0.022). Additionally, patients aged 40–49 with luminal tumors showed significantly higher CD163‐positive TAM levels than those <40 and a trend of higher levels than those aged 50–59 years (Figure 2E, *p =* 0.028 and *p =* 0.081).

### Positive PD‐L1 is associated with aggressive clinico‐pathologic features and young age

Due to limitations in tissue availability, PD‐L1 IHC staining was performed only for the BCY‐B cohort. PD‐L1 positivity correlated with clinico‐pathologic features similar to the patterns observed for the immune cell markers (CD45, CD3, CD8, FOXP3, and CD163), showing more frequent PD‐L1 positivity in ER‐ and PR‐negative tumors and associations with higher histologic grade, elevated Ki67 levels, and non‐luminal molecular subtypes (Table 3).

**Table 3:** PD‐L1 in relation to clinico‐pathologic features

	SP142 positive immune cells			SP142 positive tumor cells			22C3 positive immune cells			22C3 positive tumor cells		
	Negative	Positive	*p*	Negative	Positive	*p*	Negative	Positive	*p*	Negative	Positive	*p*
	*n* (%)	*n* (%)		*n* (%)	*n* (%)		*n* (%)	*n* (%)		*n* (%)	*n* (%)	
Age at diagnosis												
<40	56 (77.8)	16 (22.2)	0.86	58 (80.6)	14 (19.4)	0.025	60 (84.5)	11 (15.5)	0.40	57 (82.6)	12 (17.4)	0.022
40–49	167 (78.8)	45 (21.2)		191 (90.5)	20 (9.5)		182 (88.3)	24 (11.7)		190 (92.2)	16 (7.8)	
Tumor diameter												
≤2.0 cm	129 (80.6)	31 (19.4)	0.33	143 (89.4)	17 (10.6)	0.47	139 (90.8)	14 (9.2)	0.062	144 (95.4)	7 (4.6)	0.001
>2.0 cm	91 (75.8)	29 (24.2)		103 (86.6)	16 (13.4)		100 (83.3)	20 (16.7)		100 (83.3)	20 (16.7)	
Nodal status												
Negative	117 (80.7)	28 (19.3)	0.28	129 (89.6)	15 (10.4)	0.34	125 (89.3)	15 (10.7)	0.27	131 (92.9)	10 (7.1)	0.065
Positive	101 (75.4)	33 (24.6)		115 (85.8)	19 (14.2)		112 (84.8)	20 (15.2)		111 (86.0)	18 (14.0)	
Histological grade												
Low (grade 1)	50 (92.6)	4 (7.4)	0.009	53 (98.1)	1 (1.9)	0.018	54 (94.7)	3 (5.3)	0.070	57 (100.0)	0 (0.0)	0.005
High (grades 2 and 3)	165 (76.7)	50 (23.3)		186 (86.9)	28 (13.1)		176 (85.9)	29 (14.1)		178 (87.7)	25 (12.3)	
ER												
Positive	163 (85.3)	28 (14.7)	<0.001	179 (94.2)	11 (5.8)	<0.001	175 (93.6)	12 (6.4)	<0.001	176 (94.6)	10 (5.4)	<0.001
Negative	60 (64.5)	33 (35.5)		70 (75.3)	23 (24.7)		67 (74.4)	23 (25.6)		71 (79.8)	18 (20.2)	
PR												
Positive	155 (83.3)	31 (16.7)	0.007	170 (91.9)	15 (8.1)	0.005	170 (92.4)	14 (7.6)	<0.001	174 (95.1)	9 (4.9)	<0.001
Negative	68 (69.4)	30 (30.6)		79 (80.6)	19 (19.4)		72 (77.4)	21 (22.6)		73 (79.3)	19 (20.7)	
HER2												
Negative	183 (78.9)	49 (21.1)	0.65	205 (88.7)	26 (11.3)	0.35	201 (88.5)	26 (11.5)	0.13	205 (90.7)	21 (9.3)	0.21
Positive	38 (76.0)	12 (24.0)		42 (84.0)	8 (16.0)		37 (80.4)	9 (19.6)		38 (84.4)	7 (15.6)	
Ki67												
Low	116 (88.5)	15 (11.5)	<0.001	127 (97.7)	3 (2.3)	<0.001	120 (96.0)	5 (4.0)	<0.001	121 (97.6)	3 (2.4)	<0.001
High	105 (69.5)	46 (30.5)		120 (79.5)	31 (20.5)		119 (79.9)	30 (20.1)		123 (83.1)	25 (16.9)	
Molecular subtypes (IHC)												
Luminal A	100 (91.7)	9 (8.3)	<0.001	105 (97.2)	3 (2.8)	<0.001	101 (96.2)	4 (3.8)	<0.001	102 (97.1)	3 (2.9)	<0.001
Luminal B/HER2 neg	59 (75.6)	19 (24.4)		68 (87.2)	10 (12.8)		68 (88.3)	9 (11.7)		69 (90.8)	7 (9.2)	
Luminal B/HER2 pos	19 (76.0)	6 (24.0)		23 (92.0)	2 (8.0)		22 (91.7)	2 (8.3)		23 (95.8)	1 (4.2)	
HER2 positive	19 (76.0)	6 (24.0)		19 (76.0)	6 (24.0)		15 (68.2)	7 (31.8)		15 (71.4)	6 (28.6)	
Triple negative	24 (53.3)	21 (46.7)		32 (71.1)	13 (28.9)		32 (71.1)	13 (28.9)		34 (75.6)	11 (24.4)	
CK5/6												
Negative	194 (83.3)	39 (16.7)	<0.001	214 (92.2)	18 (7.8)	<0.001	203 (91.0)	20 (9.0)	<0.001	208 (93.7)	14 (6.3)	<0.001
Positive	28 (56.0)	22 (44.0)		34 (68.0)	16 (32.0)		36 (70.6)	15 (29.4)		36 (72.0)	14 (28.0)	
Basal‐like phenotype												
Absent	209 (82.0)	46 (18.0)	<0.001	232 (91.3)	22 (8.7)	<0.001	218 (89.0)	27 (11.0)	0.011	223 (91.8)	20 (8.2)	0.001
Present	13 (46.4)	15 (53.6)		16 (57.1)	12 (42.9)		21 (72.4)	8 (27.6)		21 (72.4)	8 (27.6)	

PD‐L1 evaluated by two antibodies, SP142 and 22C3, as immune or tumor positive staining in relation to clinico‐pathological features. Only data from BCY‐B cohort; young women all aged under 50 years were employed. The *p* values by Pearson's chi‐square test. Basal‐like phenotype defined as triple‐negative and CK5/6 positive. Missing data: PD‐L1 22C3: *n =* 34; PD‐L1 SP142: *n =* 36; tumor diameter: *n =* 4; lymph node status: *n =* 5; tumor grade: *n =* 15; ER: *n =* 0; PR: *n =* 0; HER2: *n =* 4; Ki67: *n =* 8; molecular subtypes: *n =* 5; CK5/6: *n =* 19; basal‐like: *n =* 21.

PD‐L1 expression was assessed using two different PD‐L1 antibodies (SP142 and 22C3). For both antibodies, younger patients presented significantly higher levels of expression of PD‐L1 positivity in tumor cells, whereas no age‐related differences were observed for PD‐L1 immune cell positivity (Figure 3B, both *p* < 0.025). Subgroup analyses revealed that this difference was more pronounced in non‐luminal tumors, reaching statistical significance for the 22C3 antibody clone only (Figure 3C, *p =* 0.045). Further stratification by molecular subtypes showed that the association persisted within the triple‐negative subgroup. Specifically, the observation remained significant for the SP142 antibody (supplementary material, Figure S4, *p =* 0.036), with a trend toward significance for the 22C3 antibody (*p =* 0.066). No significant differences were observed in other molecular subtypes (data not shown).

**Figure 3:**
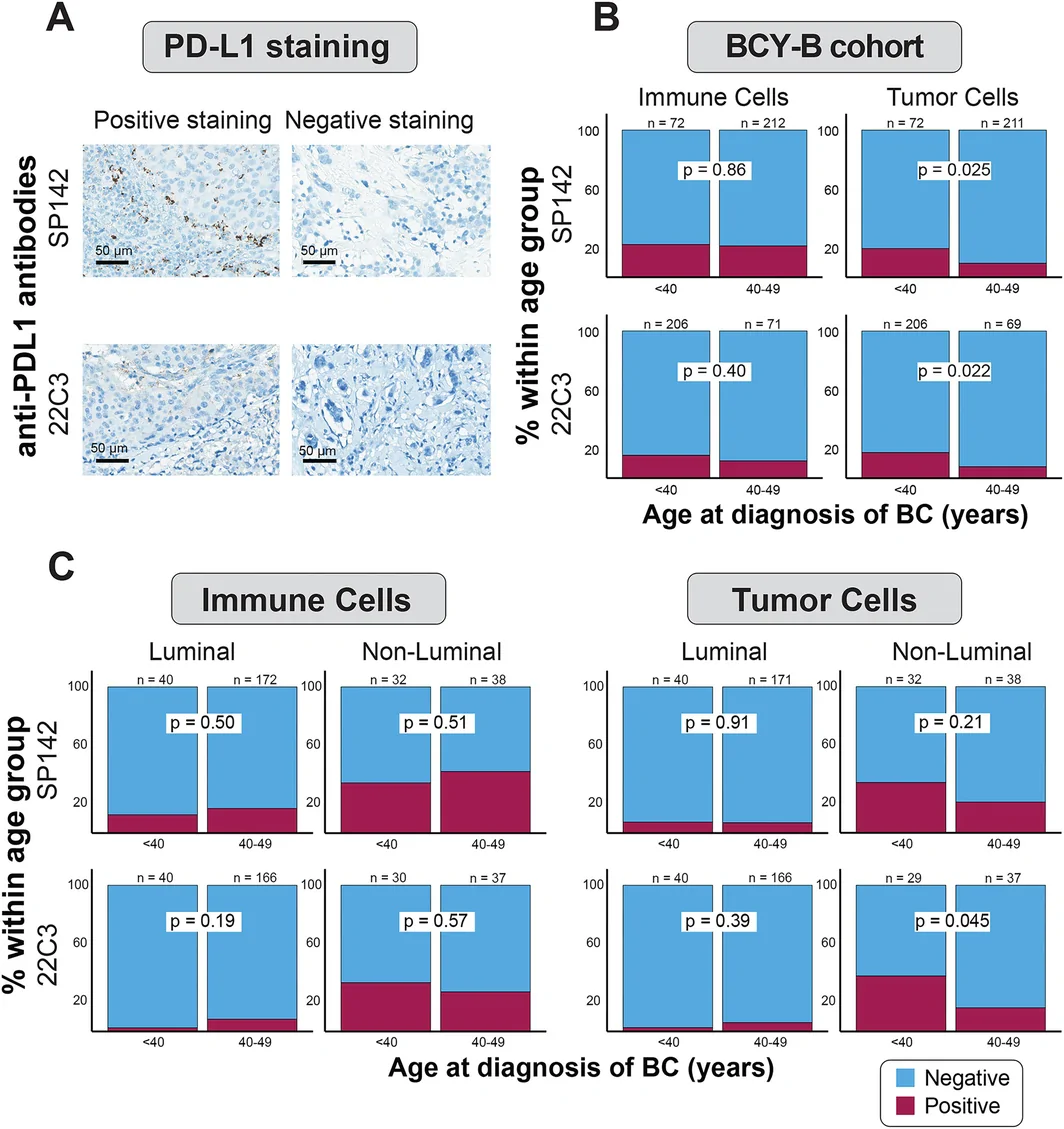
PD‐L1 staining in patients aged under 40 versus 40–49 years. (A) Immunohistochemical (IHC) evaluation of PD‐L1 (22C3 and SP142 antibodies) markers demonstrating positive staining and negative staining. All images; scale bar, 50 μm. (B, C) Evaluation of PD‐L1 using two different monoclonal antibodies (SP142 and 22C3) in the BCY‐B cohort (young; SP142 *n =* 284, 22C3 *n =* 277) across age groups (B) and luminal (SP142 *n =* 212, 22C3 *n =* 206) or non‐luminal (SP142 *n =* 70, 22C3 *n =* 67) subtypes (C). Positive staining is defined as any certain staining in the respective cells. Luminal subgroup is defined as Luminal A, Luminal B HER2‐negative, and Luminal B HER2‐positive. Non‐Luminal subgroup is defined as HER2‐positive (non‐luminal) and triple‐negative. The *p* values by Pearson chi‐square statistics.

### Prognostic value of immune cell infiltration in relation to age

We investigated the prognostic value of immune cell markers across the entire cohort and stratified by age and molecular subtypes (luminal versus non‐luminal). In the overall cohort, independent of age, CD163 was the only marker to demonstrate significant prognostic value (Figure 4A, *p =* 0.014, combined cohorts). Within age‐stratified analyses, CD163 retained significance for reduced survival only in the 50–59‐year age group (supplementary material, Figures S5 and S6; *p =* 0.039, BCY‐B and TBC cohorts separately), suggesting an age‐associated impact on survival for CD163‐positive TAMs. However, no significant interaction between age and CD163 was observed, neither when analyses were performed within the overall cohort (*p =* 0.16, missing = 32/593) nor within the luminal subgroup (*p =* 0.18; missing = 17/481).

**Figure 4:**
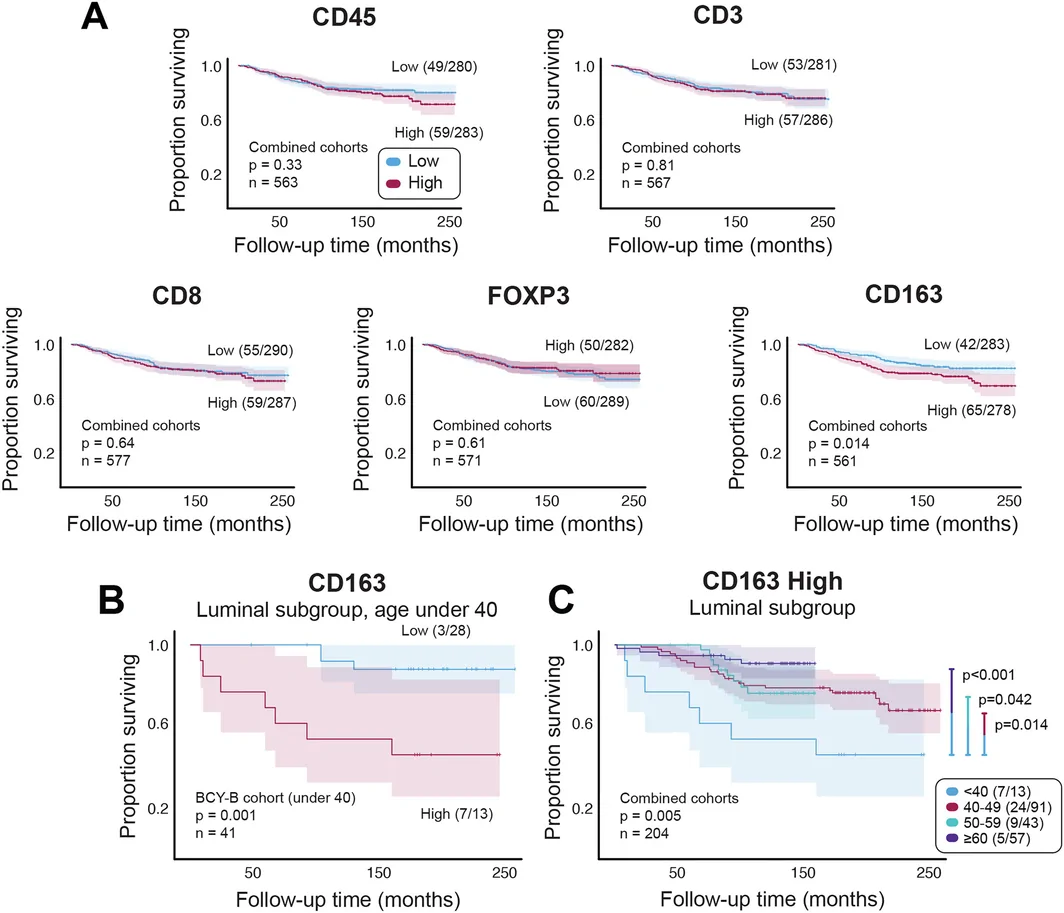
Patient survival in relation to infiltrating immune cell markers. (A) Breast cancer disease‐specific survival prospects according to immune cell marker staining of CD45 (*n =* 563, missing = 30); CD3 (*n =* 567, missing = 26), CD8 (*n =* 577, missing = 16), FOXP3 (*n =* 571, missing = 22), and CD163 (*n =* 561, missing = 32), employing the combined BCY‐B and TBC cohorts. (B) Breast cancer‐specific survival in patients aged under 40 years according to high/low CD163 staining (*n =* 41, missing = 9). (C) Breast cancer‐specific survival of patients with high CD163 across all age groups in luminal subtypes (*n =* 204, missing = 17). Kaplan–Meier survival analysis was applied with log‐rank test for difference. Luminal subgroup is defined as Luminal A, Luminal B HER2‐negative, and Luminal B HER2‐positive. All markers are dichotomized by the median.

When stratifying for molecular subtypes, high levels of CD3‐positive TILs were significantly associated with improved survival in the non‐luminal subgroup (*p =* 0.012), while CD8‐positive TILs showed a trend toward positive prognostic value (supplementary material, Figure S7, *p =* 0.067, combined cohorts). High levels of CD163‐positive macrophages were associated with poorer survival in the luminal subgroup (supplementary material, Figure S7, *p =* 0.009, combined cohorts). There was a significant interaction between CD163 and subgroups (*p =* 0.018; missing = 37/593), indicating a stronger prognostic value of CD163 in the luminal subgroup.

Notably, within the luminal subgroup, CD163 demonstrated strong prognostic value in patients under 40 years of age within the luminal subgroup (Figure 4B, *p =* 0.001, BCY‐B cohort). Furthermore, among tumors with high levels of CD163 positive macrophages, those under 40 years exhibited significantly shorter survival compared to the older groups (Figure 4C, *p =* 0.005, combined cohorts). Finally, PD‐L1 expression did not demonstrate significant prognostic value in any of the survival analyses conducted, regardless of age group or molecular subtype (not shown).

## Discussion

Younger age is associated with more active immune responses, but its impact on immune infiltration in breast cancer remains unclear [36]. Given the immune system's dual role in tumor suppression and promotion, comprehensive profiling is needed to understand age‐related immune differences in breast cancer.

In this study, we used two in‐house cohorts, representing younger and older BC patients, investigating potential age‐related variations in tumor immune cell infiltration across molecular subtypes. Overall, the differences were modest and general TIL markers (CD45, CD3) showed no significant age differences, although younger patients (<50) presented with higher levels of CD163‐positive TAMs and lower levels of FOXP3‐positive TILs. Among patients <50 years, those under 40 exhibited more frequent PD‐L1 expression on tumor cells. Previous studies have reported higher levels of immune cells in BC tumors from younger patients [25, 26, 27]. TIL levels remained stable across age groups, except for FOXP3‐positive TILs, which were significantly increased in patients aged ≥50 years. This finding could be consistent with previous reports of age‐associated Treg accumulation and immunosenescence [24, 37, 38]. Higher levels of FOXP3‐positive TILs in patients aged 50 years and older were observed in both luminal and non‐luminal groups, indicating an age‐related biological effect, partly independent of tumor subtype. However, it should be noted that FOXP3 IHC does not allow distinction between suppressive Tregs and activated conventional T‐cells expressing FOXP3 [39]. Also, the nature of age‐related Treg accumulation – functional versus senescent – remains debated, with inconsistent findings due to methodological differences [40].

Our observations suggest that mechanisms beyond immune cell recruitment may contribute to immune evasion of tumors in young patients. A study reported reduced expression of CD134 (OX‐40), a co‐stimulatory molecule for T‐helper cells, in young patients, and spatial differences of this protein by age – potentially reflecting differences in immune activation or suppression [26].

Notably, we found more frequent PD‐L1 positivity on tumor cells from patients <40 years when compared to those aged 40–49, consistent with results from prior studies in early‐stage BC [41]. However, clinical evidence regarding age‐associated differences in immune checkpoint inhibitors (ICIs) sensitivity remains limited. Further research looking into the age‐specific differences in immune checkpoint pathways activation and response to ICI in the clinical setting is warranted. Conversely, studies in other malignancies, including malignant melanoma and non‐small cell lung carcinoma, indicate that ICI therapy may be effective across age groups, while some reports suggest a greater benefit in patients aged >65 years [42, 43, 44]. To our knowledge, none of the prior studies have applied cut‐points at 40 or 50 years, comparable to the age groups in our study.

Among the immune markers assessed, CD163 was the only marker demonstrating significant prognostic value in the overall cohort. Consistent with previous reports, high levels of CD163‐positive TAMs were associated with poorer prognosis. However, prior studies have noted subgroup‐dependent differences, with less consistent findings in triple‐negative cases [45, 46, 47]. In our subgroup analyses, elevated levels of CD163 positive TAMs were particularly associated with poorer survival especially in patients with luminal BC. Conversely, in non‐luminal tumors, higher levels of CD3 positive TILs were linked to improved survival, and higher levels of CD8 showed a trend toward better survival. FOXP3 immune cell markers showed no prognostic relevance. Based on findings from a large multicohort study by Ali *et al*, we anticipated a positive prognostic value for CD8 positive TILs in both luminal and non‐luminal subtypes [48]. Our results align with this finding in patients with non‐luminal tumors but in the luminal tumors, in line with observations by Liu *et al*, who reported a stronger prognostic role of CD8 positive TILs in non‐luminal BC [49]. FOXP3 positive regulatory T cells demonstrated no prognostic value in our cohort, although their role in prognostication remains controversial. While high levels of FOXP3 positive immune cells are generally associated with poorer outcome, some studies suggest a favorable prognostic effect in ER‐negative tumors [50, 51, 52, 53].

Of note, age stratification of the immune markers did not provide definitive prognostic value, possibly due to limited number of cases. However, a recent study by Tesch *et al* reported prognostic significance in young women (≤40 years) with hormone receptor positive/HER2 negative tumors [29]. Further, the same study demonstrated that non‐CD8 T cells, Tregs, and PD‐L1 positive TILs within the tumor compartment were associated with improved breast cancer‐free and distant disease‐free survival [29]. In contrast, overall survival was primarily influenced by cytotoxic T cells [29]. Taken together, these findings suggest that TILs may have a prognostic value in younger patients; however, this effect does not appear to differ substantially between younger and older age groups. The use of two cohorts and different observers could potentially introduce bias and necessitated harmonization of data. Nevertheless, inter‐rater reliability was robust, as demonstrated by strong Kappa statistics and ranked intraclass correlation coefficients. Moreover, appropriate adjustments were applied to mitigate subjectivity of the scoring and to ensure consistency across assessments.

Our retrospective design allowed for a long follow‐up period, which is particularly valuable in the BC setting. However, it also has limitations, including the ability to capture differences between calendar periods, geographic source, screening context, tissue processing, observers, and treatment. The observer differences were accounted for in the harmonization of cohorts. However, due to limited availability, we were unable to adjust for the remaining differences. BC treatment evolved during the sequential acquisition of the two cohorts (BCY‐B, 1996–2003; and TBC, 2004–2009). While no major changes occurred in endocrine therapy during this period, the use of adjuvant chemotherapy increased gradually, and hence, the introduction of Trastuzumab (Herceptin) in 2006 represents the most substantial treatment‐related difference between the cohorts. TILs serve as both positive prognostic and predictive biomarkers for anti‐HER2 therapy in BC and could be linked to better survival for HER2‐patients in our study, although this would apply only to a small subset of patients (*n =* 14) [54].

PD‐L1 staining was not available from the older TBC cohort. Therefore, the present age‐related PD‐L1 analyses reflect differences using 40 years as a cut‐off within the younger BCY‐B cohort (under 50 years). Future studies should explore PD‐L1 expression also across older patients to investigate the broader age‐related differences in context.

Additionally, our study was hampered by the relatively small sample size, hindering more granular analyses within each molecular subgroup. Furthermore, the availability of additional clinical and tumor‐related variables, such as treatment, menopausal status, and spatial distribution of immune cells within the tumor microenvironment, would have strengthened the analyses. Lastly, we explored several individual immune markers across age groups and clinico‐pathological variables, including molecular subgroups. This approach involves numerous statistical comparisons; some findings may be attributable to chance and should therefore be considered exploratory. Validation in independent cohorts, with analyses focused on specific immune cell populations within defined patient subgroups, is warranted. Although our primary focus was on age‐related variation in tumor biology, particularly immune responses, it is important to acknowledge that systemic immune aging, independent of tumor characteristics, may also modulate tumor–host interactions and influence immune responses and effects. These intertwined mechanisms introduce additional complexity to the interpretation of immune infiltration patterns and their clinical relevance. In summary, our results underscore the heterogeneous landscape of TIL markers across BC subtypes and their prognostic implications, with only limited differences observed across age groups. Further subtype‐specific and age‐stratified investigations are warranted, particularly in younger patients, to address the current knowledge gaps and advance immune biomarker‐guided treatment strategies.

## Author contributions statement

ROCH, LAA, EW and EAH contributed to the study design, performed the statistical analyses and wrote the manuscript. ROCH and IW performed the immunohistochemistry laboratory work. ROCH, YC, TAK, AKMS, AAS, UH, ABK, AFT, IW, TA, AH, LAA, EW and EAH contributed to data collection, interpretation of the results, provided critical input and participated in the review and editing of the manuscript. All authors read and approved the final manuscript.

## Supporting information


**Figure S1.** Study flowchart
**Figure S2.** Sensitivity analysis of cohort harmonization
**Figure S3.** Sensitivity analysis and different Ki67 cut points and effect on subtype distribution and survival
**Figure S4.** PD‐L1 staining in patients aged under 40 versus 40–49 years, for the triple‐negative subtype only
**Figure S5.** Infiltrating immune cells and patient survival – across age groups
**Figure S6.** Infiltrating immune cells and hazard ratio
**Figure S7.** Infiltrating immune cells and patient survival – across subgroups
**Table S1.** Descriptive information on the inter‐observer dataset
**Table S2.** Ki67 sensitivity analyses
**Table S3.** Clinico‐pathologic features and infiltrating immune cells

## Data Availability

The data that support the findings of this study are available from the corresponding author upon reasonable request.

## References

[1] Leclère B , Molinié F , Trétarre B , *et al*. Trends in incidence of breast cancer among women under 40 in seven European countries: a GRELL cooperative study. Cancer Epidemiol 2013; 37: 544–549. 10.1016/j.canep.2013.05.001 23768969

[2] Xu S , Murtagh S , Han Y , *et al*. Breast cancer incidence among US women aged 20 to 49 years by race, stage, and hormone receptor status. JAMA Netw Open 2024; 7: e2353331. 10.1001/jamanetworkopen.2023.53331 PMC10818222 38277147

[3] Fredholm H , Magnusson K , Lindström LS , *et al*. Long‐term outcome in young women with breast cancer: a population‐based study. Breast Cancer Res Treat 2016; 160: 131–143. 10.1007/s10549-016-3983-9 PMC5050247 27624330

[4] Anders CK , Hsu DS , Broadwater G , *et al*. Young age at diagnosis correlates with worse prognosis and defines a subset of breast cancers with shared patterns of gene expression. J Clin Oncol 2008; 26: 3324–3330. 10.1200/JCO.2007.14.2471 18612148

[5] Zhu JW , Charkhchi P , Adekunte S , *et al*. What is known about breast cancer in young women? Cancer 2023; 15: 1917. 10.3390/cancers15061917 PMC10047861 36980802

[6] Morrison DH , Rahardja D , King E , *et al*. Tumour biomarker expression relative to age and molecular subtypes of invasive breast cancer. Br J Cancer 2012; 107: 382–387. 10.1038/bjc.2012.219 PMC3394967 22713661

[7] Loi S , Sirtaine N , Piette F , *et al*. Prognostic and predictive value of tumor‐infiltrating lymphocytes in a phase III randomized adjuvant breast cancer trial in node‐positive breast cancer comparing the addition of docetaxel to doxorubicin with doxorubicin‐based chemotherapy: BIG 02‐98. J Clin Oncol 2013; 31: 860–867. 10.1200/JCO.2011.41.0902 23341518

[8] Svanøe AA , Humlevik ROC , Knutsvik G , *et al*. Age‐related phenotypes in breast cancer: a population‐based study. Int J Cancer 2024; 154: 2014–2024. 10.1002/ijc.34863 38319154

[9] DeNardo DG , Coussens LM . Inflammation and breast cancer. Balancing immune response: crosstalk between adaptive and innate immune cells during breast cancer progression. Breast Cancer Res 2007; 9: 212. 10.1186/bcr1746 PMC2206719 17705880

[10] Salgado R , Denkert C , Demaria S , *et al*. The evaluation of tumor‐infiltrating lymphocytes (TILs) in breast cancer: recommendations by an International TILs Working Group 2014. Ann Oncol 2015; 26: 259–271. 10.1093/annonc/mdu450 PMC6267863 25214542

[11] Loi S , Michiels S , Salgado R , *et al*. Tumor infiltrating lymphocytes are prognostic in triple negative breast cancer and predictive for trastuzumab benefit in early breast cancer: results from the FinHER trial. Ann Oncol 2014; 25: 1544–1550. 10.1093/annonc/mdu112 24608200

[12] Denkert C , von Minckwitz G , Darb‐Esfahani S , *et al*. Tumour‐infiltrating lymphocytes and prognosis in different subtypes of breast cancer: a pooled analysis of 3771 patients treated with neoadjuvant therapy. Lancet Oncol 2018; 19: 40–50. 10.1016/S1470-2045(17)30904-X 29233559

[13] Li S , Zhang Y , Zhang P , *et al*. Predictive and prognostic values of tumor infiltrating lymphocytes in breast cancers treated with neoadjuvant chemotherapy: a meta‐analysis. Breast 2022; 66: 97–109. 10.1016/j.breast.2022.10.001 PMC9550538 36219945

[14] Xu T , Zhang H , Yang BB , *et al*. Tumor‐infiltrating immune cells state‐implications for various breast cancer subtypes. Front Immunol 2025; 16: 1550003. 10.3389/fimmu.2025.1550003 PMC12116345 40438111

[15] Trowbridge IS , Thomas ML . CD45: an emerging role as a protein tyrosine phosphatase required for lymphocyte activation and development. Annu Rev Immunol 1994; 12: 85–116. 10.1146/annurev.iy.12.040194.000505 8011300

[16] Wang Y , Becker D , Vass T , *et al*. A conserved CXXC motif in CD3ε is critical for T cell development and TCR signaling. PLoS Biol 2009; 7: e1000253. 10.1371/journal.pbio.1000253 PMC2776832 19956738

[17] Mahmoud SMA , Paish EC , Powe DG , *et al*. Tumor‐infiltrating CD8+ lymphocytes predict clinical outcome in breast cancer. J Clin Oncol 2011; 29: 1949–1955. 10.1200/JCO.2010.30.5037 21483002

[18] Lu L , Barbi J , Pan F . The regulation of immune tolerance by FOXP3. Nat Rev Immunol 2017; 17: 703–717. 10.1038/nri.2017.75 PMC5793224 28757603

[19] Heusinkveld M , van der Burg SH . Identification and manipulation of tumor associated macrophages in human cancers. J Transl Med 2011; 9: 216. 10.1186/1479-5876-9-216 PMC3286485 22176642

[20] He X , Xu C . Immune checkpoint signaling and cancer immunotherapy. Cell Res 2020; 30: 660–669. 10.1038/s41422-020-0343-4 PMC7395714 32467592

[21] Corti C , Koca B , Rahman T , *et al*. Recent advances in immune checkpoint inhibitors for triple‐negative breast cancer. Immunotargets Ther 2025; 14: 339–357. 10.2147/ITT.S495751 PMC11974553 40196378

[22] Cortes J , Rugo HS , Cescon DW , *et al*. Pembrolizumab plus chemotherapy in advanced triple‐negative breast cancer. N Engl J Med 2022; 387: 217–226. 10.1056/NEJMoa2202809 35857659

[23] Schmid P , Adams S , Rugo HS , *et al*. Atezolizumab and nab‐paclitaxel in advanced triple‐negative breast cancer. N Engl J Med 2018; 379: 2108–2121. 10.1056/NEJMoa1809615 30345906

[24] Sayed S , Koka H , Abubakar M , *et al*. Tumour infiltrating lymphocytes (TILs) and immune composition in breast cancer patients from Kenya: spatial distributions and associations with risk factors and tumour characteristics. Breast Cancer Res Treat 2023; 199: 401–413. 10.1007/s10549-023-06921-3 PMC11568797 37010652

[25] Takada K , Kashiwagi S , Asano Y , *et al*. Differences in tumor‐infiltrating lymphocyte density and prognostic factors for breast cancer by patient age. World J Surg Oncol 2022; 20: 38. 10.1186/s12957-022-02513-5 PMC8851811 35177074

[26] Hatse S , Lambrechts Y , Antoranz Martinez A , *et al*. Dissecting the immune infiltrate of primary luminal B‐like breast carcinomas in relation to age. J Pathol 2024; 264: 344–356. 10.1002/path.6354 39344093

[27] Berben L , Floris G , Kenis C , *et al*. Age‐related remodelling of the blood immunological portrait and the local tumor immune response in patients with luminal breast cancer. Clin Transl Immunol 2020; 9: e1184. 10.1002/cti2.1184 PMC7532981 33024560

[28] Chumsri S , Serie DJ , Li Z , *et al*. Effects of age and immune landscape on outcome in HER2‐positive breast cancer in the NCCTG N9831 (alliance) and NSABP B‐31 (NRG) trials. Clin Cancer Res 2019; 25: 4422–4430. 10.1158/1078-0432.CCR-18-2206 PMC6634998 30808774

[29] Tesch ME , Zheng Y , Guzman‐Arocho YD , *et al*. Association of tumor‐infiltrating lymphocyte subtypes with clinical characteristics and prognosis in young women with hormone receptor‐positive breast cancer. Clin Cancer Res 2025; 32: 2268–2278. 10.1158/1078-0432.CCR-25-0948 41117836

[30] de Jong VMT , Wang Y , Ter Hoeve ND , *et al*. Prognostic value of stromal tumor‐infiltrating lymphocytes in young, node‐negative, triple‐negative breast cancer patients who did not receive (neo)adjuvant systemic therapy. J Clin Oncol 2022; 40: 2361–2374. 10.1200/JCO.21.01536 PMC9287283 35353548

[31] Chen Y , Klingen TA , Aas H , *et al*. Tumor‐associated lymphocytes and macrophages are related to stromal elastosis and vascular invasion in breast cancer. J Pathol Clin Res 2021; 7: 517–527. 10.1002/cjp2.226 PMC8363927 34076969

[32] Vennapusa B , Baker B , Kowanetz M , *et al*. Development of a PD‐L1 complementary diagnostic immunohistochemistry assay (SP142) for atezolizumab. Appl Immunohistochem Mol Morphol 2019; 27: 92–100. 10.1097/PAI.0000000000000594 PMC6369970 29346180

[33] Nanda R , Chow LQM , Dees EC , *et al*. Pembrolizumab in patients with advanced triple‐negative breast cancer: phase Ib KEYNOTE‐012 study. J Clin Oncol 2016; 34: 2460–2467. 10.1200/JCO.2015.64.8931 PMC6816000 27138582

[34] Goldhirsch A , Winer EP , Coates AS , *et al*. Personalizing the treatment of women with early breast cancer: highlights of the St Gallen International Expert Consensus on the Primary Therapy of Early Breast Cancer 2013. Ann Oncol 2013; 24: 2206–2223. 10.1093/annonc/mdt303 PMC3755334 23917950

[35] Cheang MC , Cheang MCU , Chia SK , *et al*. Ki67 index, HER2 status, and prognosis of patients with luminal B breast cancer. J Natl Cancer Inst 2009; 101: 736–750. 10.1093/jnci/djp082 PMC2684553 19436038

[36] Liu Z , Liang Q , Ren Y , *et al*. Immunosenescence: molecular mechanisms and diseases. Signal Transduct Target Ther 2023; 8: 200. 10.1038/s41392-023-01451-2 PMC10182360 37179335

[37] Lages CS , Suffia I , Velilla PA , *et al*. Functional regulatory T cells accumulate in aged hosts and promote chronic infectious disease reactivation. J Immunol 2008; 181: 1835–1848. 10.4049/jimmunol.181.3.1835 PMC2587319 18641321

[38] Lumeng CN , Liu J , Geletka L , *et al*. Aging is associated with an increase in T cells and inflammatory macrophages in visceral adipose tissue. J Immunol 2011; 187: 6208–6216. 10.4049/jimmunol.1102188 PMC3237772 22075699

[39] Klein S , Kretz CC , Krammer PH , *et al*. CD127(low/−) and FoxP3(+) expression levels characterize different regulatory T‐cell populations in human peripheral blood. J Investig Dermatol 2010; 130: 492–499. 10.1038/jid.2009.313 19940860

[40] Palatella M , Guillaume SM , Linterman MA , *et al*. The dark side of Tregs during aging. Front Immunol 2022; 13: 940705. 10.3389/fimmu.2022.940705 PMC9398463 36016952

[41] Okabe M , Toh U , Iwakuma N , *et al*. Predictive factors of the tumor immunological microenvironment for long‐term follow‐up in early stage breast cancer. Cancer Sci 2017; 108: 81–90. 10.1111/cas.13114 PMC5276839 27801993

[42] Betof AS , Nipp RD , Giobbie‐Hurder A , *et al*. Impact of age on outcomes with immunotherapy for patients with melanoma. Oncologist 2017; 22: 963–971. 10.1634/theoncologist.2016-0450 PMC5553960 28476944

[43] Ellington TD , Henley SJ , Wilson RJ , *et al*. Trends in breast cancer mortality by race/ethnicity, age, and US census region, United States horizontal line 1999–2020. Cancer 2022; 129: 32–38. 10.1002/cncr.34503 PMC10128100 36309838

[44] Wu Q , Wang Q , Tang X , *et al*. Correlation between patients' age and cancer immunotherapy efficacy. Onco Targets Ther 2019; 8: e1568810. 10.1080/2162402X.2019.1568810 PMC6422380 30906662

[45] Yang M , Li Z , Ren M , *et al*. Stromal infiltration of tumor‐associated macrophages conferring poor prognosis of patients with basal‐like breast carcinoma. J Cancer 2018; 9: 2308–2316. 10.7150/jca.25155 PMC6036715 30026826

[46] Jääskeläinen MM , Tumelius R , Hämäläinen K , *et al*. High numbers of CD163+ tumor‐associated macrophages predict poor prognosis in HER2+ breast cancer. Cancer 2024; 16: 634. 10.3390/cancers16030634 PMC10854814 38339385

[47] Medrek C , Pontén F , Jirström K , *et al*. The presence of tumor associated macrophages in tumor stroma as a prognostic marker for breast cancer patients. BMC Cancer 2012; 12: 306. 10.1186/1471-2407-12-306 PMC3414782 22824040

[48] Ali HR , Provenzano E , Dawson SJ , *et al*. Association between CD8+ T‐cell infiltration and breast cancer survival in 12,439 patients. Ann Oncol 2014; 25: 1536–1543. 10.1093/annonc/mdu191 24915873

[49] Liu S , Lachapelle J , Leung S , *et al*. CD8+ lymphocyte infiltration is an independent favorable prognostic indicator in basal‐like breast cancer. Breast Cancer Res 2012; 14: R48. 10.1186/bcr3148 PMC3446382 22420471

[50] Yan M , Jene N , Byrne D , *et al*. Recruitment of regulatory T cells is correlated with hypoxia‐induced CXCR4 expression, and is associated with poor prognosis in basal‐like breast cancers. Breast Cancer Res 2011; 13: R47. 10.1186/bcr2869 PMC3219210 21521526

[51] West NR , Kost SE , Martin SD , *et al*. Tumour‐infiltrating FOXP3(+) lymphocytes are associated with cytotoxic immune responses and good clinical outcome in oestrogen receptor‐negative breast cancer. Br J Cancer 2013; 108: 155–162. 10.1038/bjc.2012.524 PMC3553524 23169287

[52] Shang B , Liu Y , Jiang S‐j , *et al*. Prognostic value of tumor‐infiltrating FoxP3+ regulatory T cells in cancers: a systematic review and meta‐analysis. Sci Rep 2015; 5: 15179. 10.1038/srep15179 PMC4604472 26462617

[53] Tian W , Wang L , Yuan L , *et al*. A prognostic risk model for patients with triple negative breast cancer based on stromal natural killer cells, tumor‐associated macrophages and growth‐arrest specific protein 6. Cancer Sci 2016; 107: 882–889. 10.1111/cas.12964 PMC4946705 27145494

[54] Luque M , Sanz‐Álvarez M , Morales‐Gallego M , *et al*. Tumor‐infiltrating lymphocytes and immune response in HER2‐positive breast cancer. Cancer 2022; 14: 6034. 10.3390/cancers14246034 PMC9776701 36551522

