# Supplementary material for: Age‐associated differences in the breast cancer immune microenvironment
Source: J Pathol Clin Res. 2026 Nov 3;12(6):e70115. doi: 10.1002/2056-4538.70115 (PMC13729828; doi:10.1002/2056-4538.70115)
Supplement: Supplementary file 1 [file CJP2-12-e70115-s001.pdf]

Age-associated differences in the breast cancer immune microenvironment

ROC Humlevik, Y Chen, TA Klingen *et al. J Pathol Clin Res.* https://doi.org/10.1002/2056-4538.70115

Supplementary Figures S1–S7
Supplementary Tables S1–S3

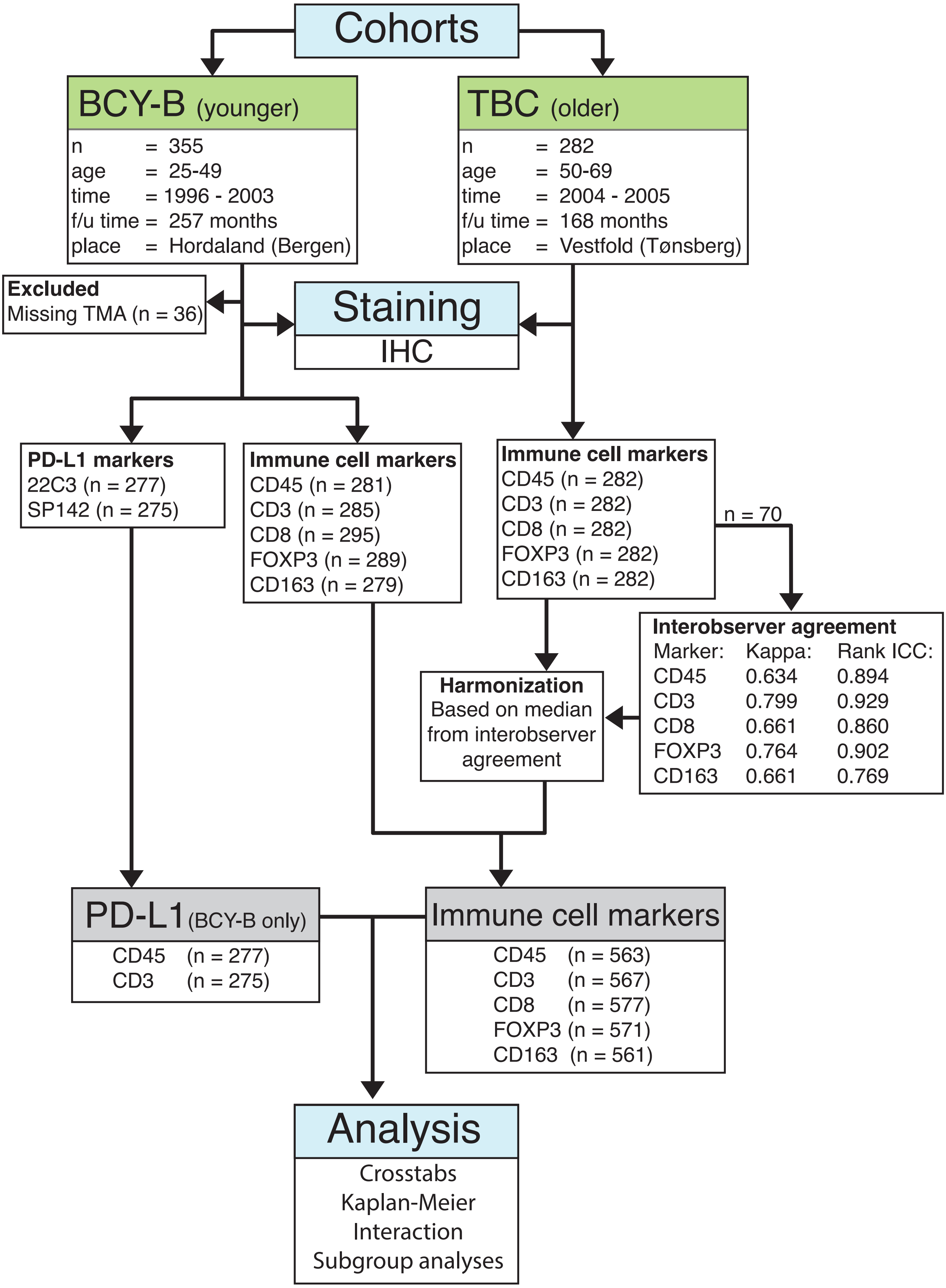

Figure S1. Study Flowchart. Study flowchart showing overview of cohorts, markers applied, assessment and analysis approach. The BCY-B (Bergen, *n* = 355) cohort consists of younger patients, while the TBC cohort (Tønsberg, *n* = 282) consist of older patients.

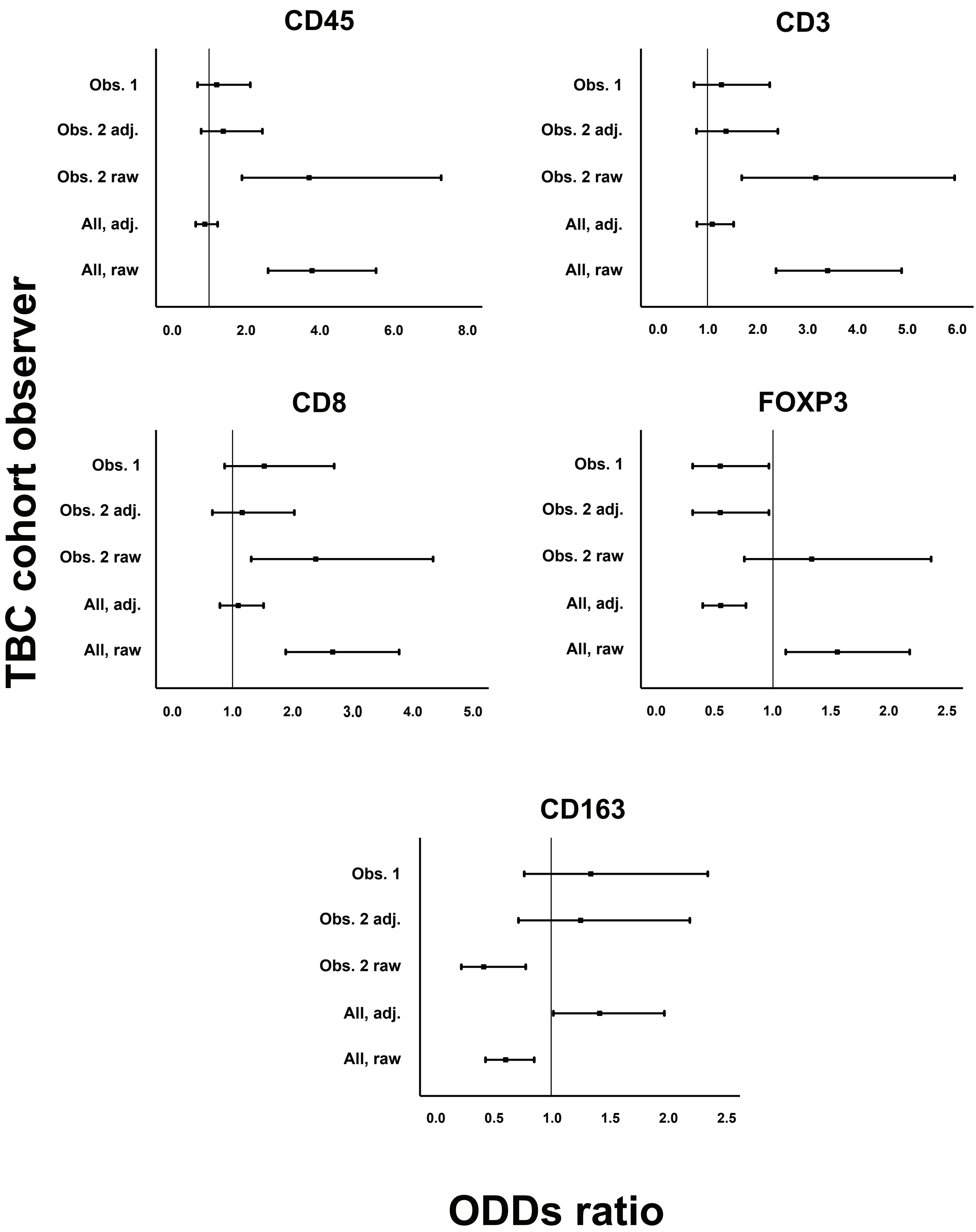

Figure S2. Sensitivity analysis of cohort harmonization. Visualization of data harmonization with a forest plot demonstrating ORs with 95% confidence intervals. ORs were calculated using low immune cell count and old age as the reference group. ORs represent the odds of having a high immune cell count in younger patients compared with older patients. The three groups represent patients from the inter-observer agreement; all are compared with the BCY-B cohort. "Obs. 1" represents counting from ROCH, the observer of BCY-B cohort. "Obs. 2 raw" represents counting from YC, the observer of the TBC cohort, before adjustments. "Obs. 2 adj." represents counting from YC after adjustments. Counts are standardized to mm^2. N (from inter-observer agreement) = 59 for all markers.

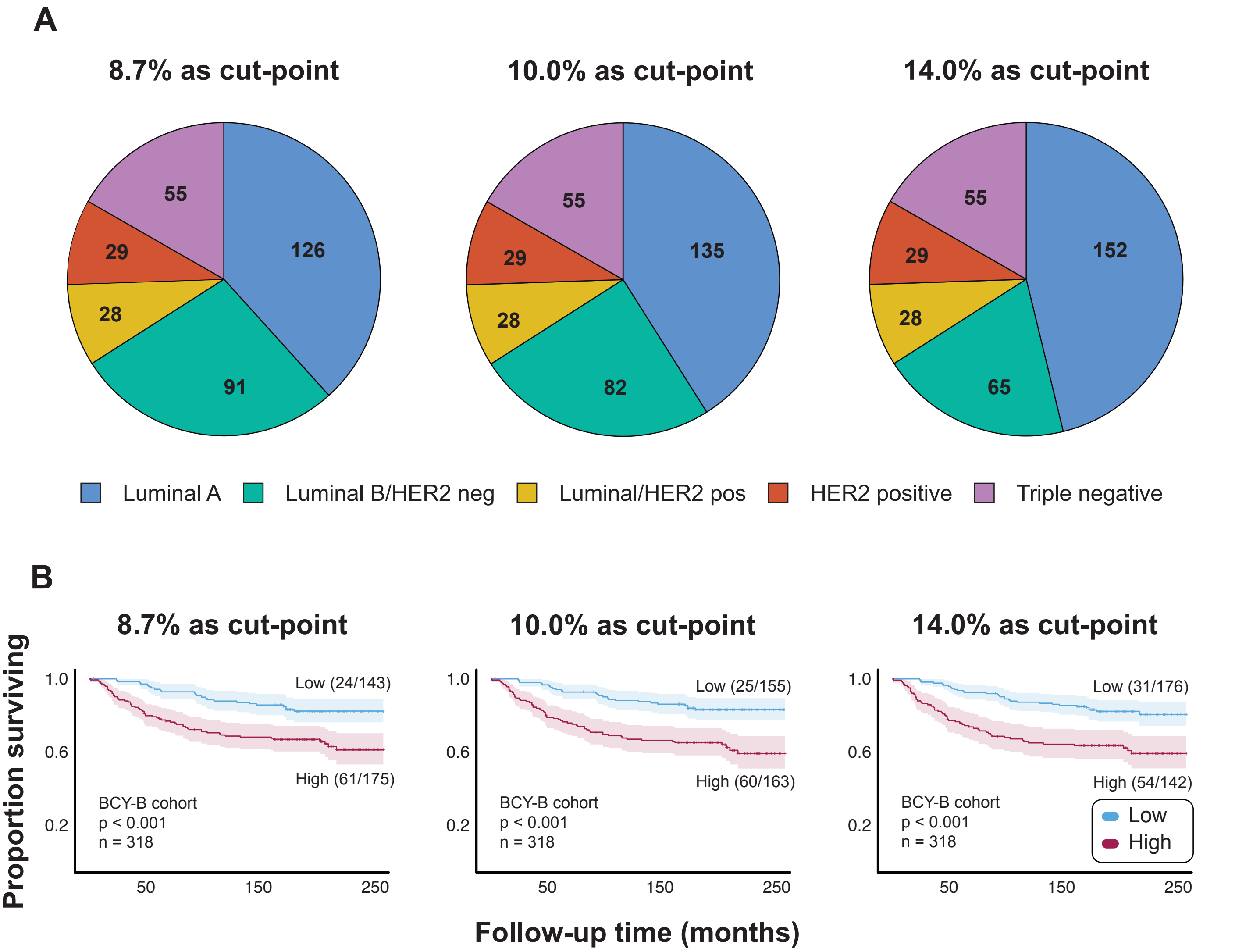

Figure S3. Sensitivity analysis and different Ki67 cut-points and effect on subtype distribution and survival. Sensitivity analyses comparing subtype allocations and survival outcomes using Ki67 cut-points of 8.7%, 10% and 14% revealed almost no difference in survival. The 8.7% cut-point was used as the reference in the analyses. (A) Molecular subgroups across the different Ki67 cut-points. (B) Kaplan-Meier plots with different Ki67 cut-points. Log-rank test for difference and breast cancer disease-specific survival as endpoint. BCY-B cohort (*n* = 355). Missing: Ki67: *n* = 26.

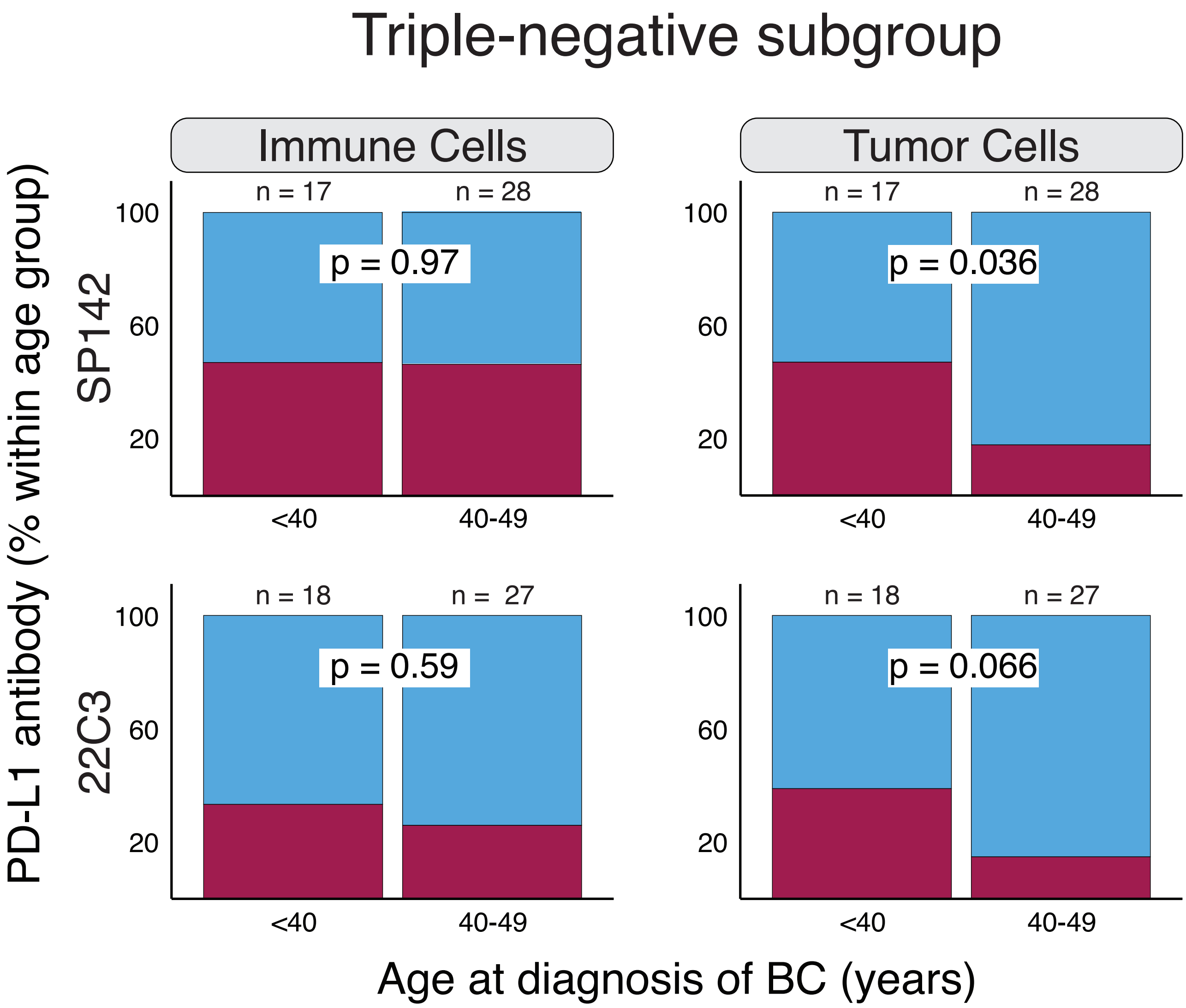

Figure S4. PD-L1 staining in patients aged under 40 versus 40–49 years, for the Triple-Negative subtype only. Evaluation of PD-L1 using two different antibodies (SP142 and 22C3) in the BCY-B cohort (young; *n* = 311) across age groups for the triple-negative subtype only. Markers are dichotomized by the median value. *P*-values by Pearson Chi-Square statistics.

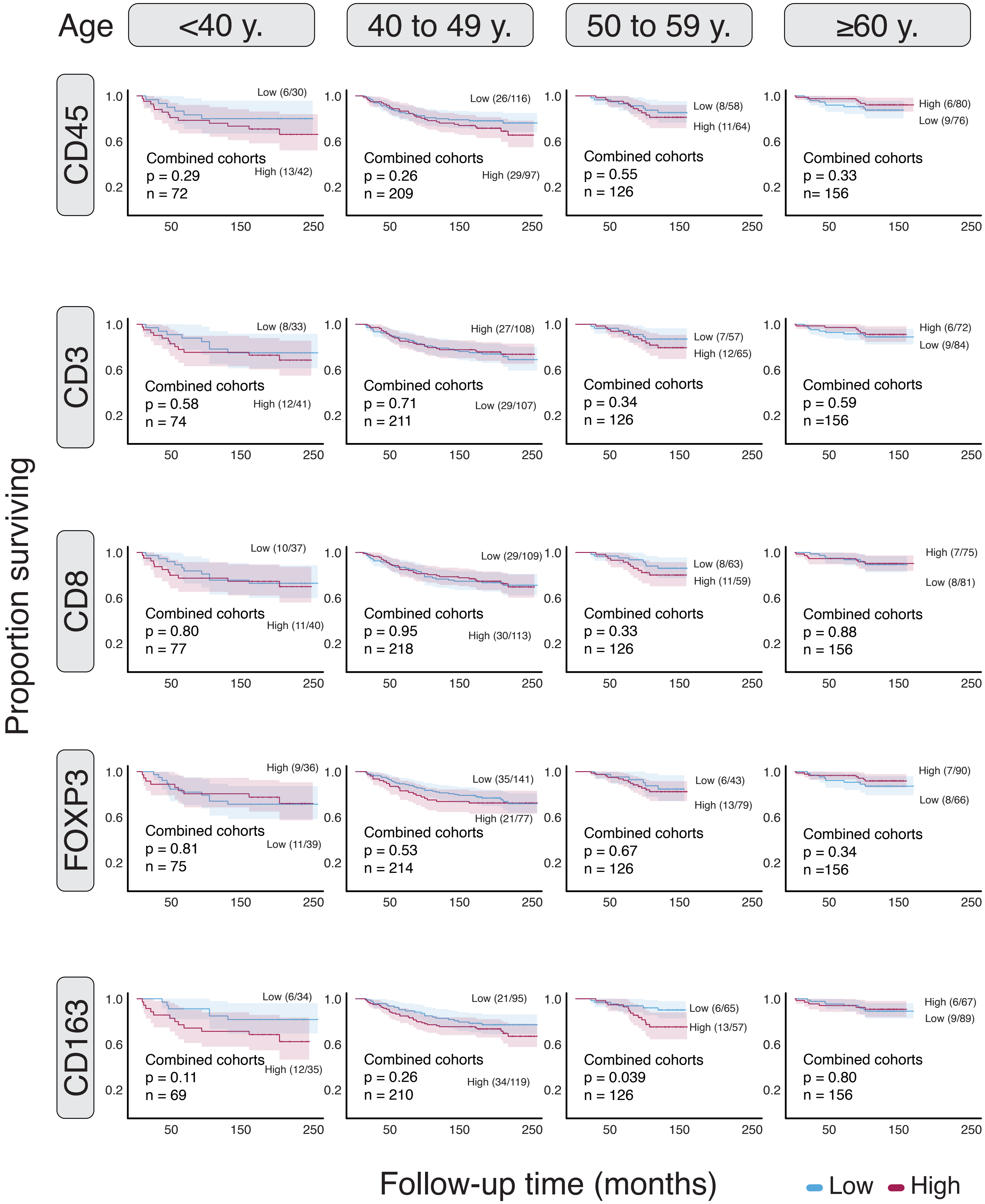

Figure S5. Infiltrating immune cells and patient survival - across age groups. Breast cancer disease-specific survival according to age at diagnosis and immune cell marker staining of CD45; CD3, CD8, FOXP3 and CD163. All markers are dichotomized by the median. Kaplan-Meier survival analysis by log-rank test for difference. Missing data: CD45: *n* = 30; CD3:*n* = 26; CD8: *n* = 16; FOXP3: *n* = 22; CD163: *n* = 32.

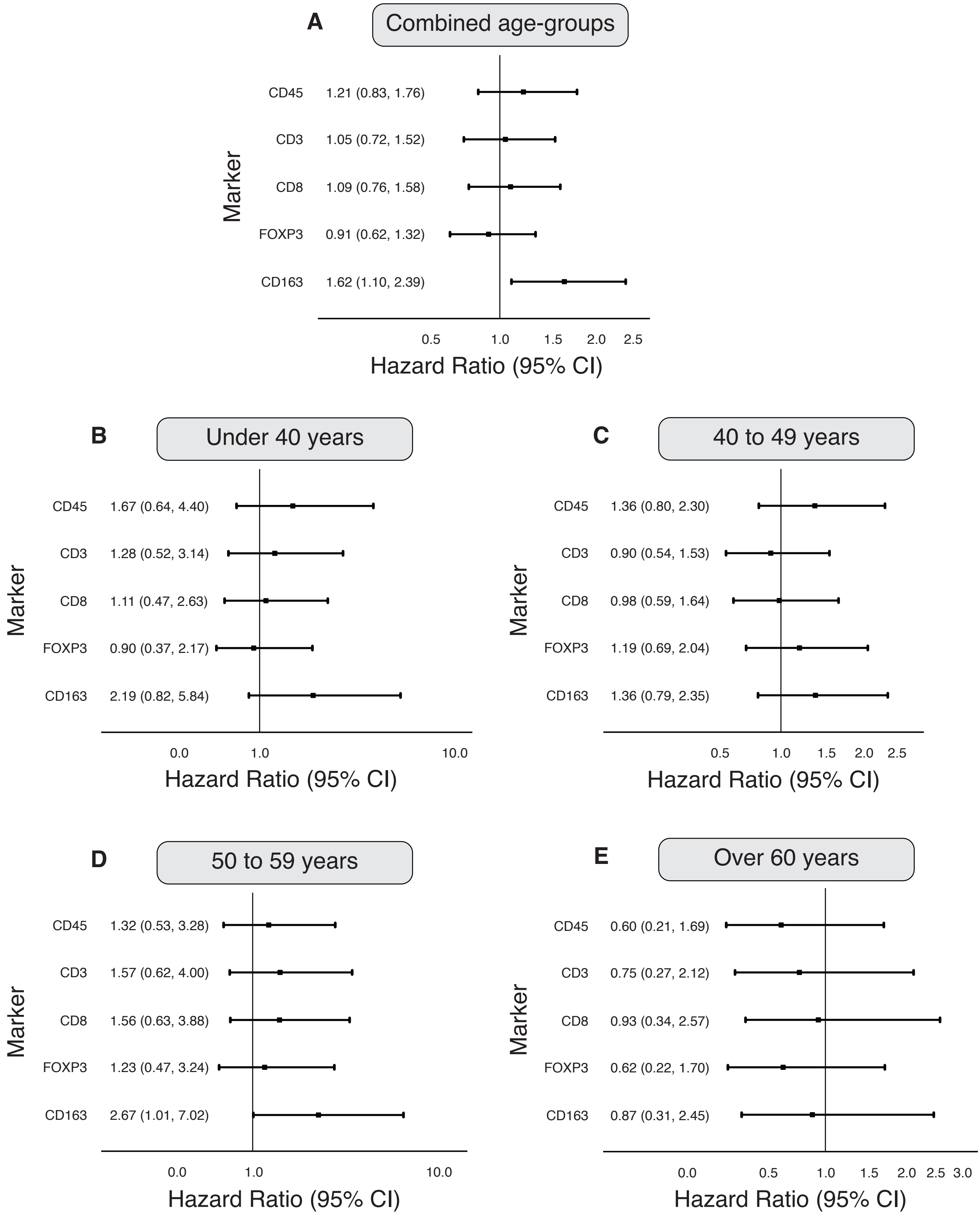

Figure S6. Infiltrating immune cells and Hazard Ratio. A forest plot showing hazard ratio with 95% confidence intervals, with breast cancer disease-specific survival as endpoint within age groups. Values are obtained from univariable cox regression analyses with immune cell marker staining of CD45, CD3, CD8, FOXP3 and CD163. All markers are dichotomized by the median. High expression is the exposed group. (A) All age-groups combined (*n* = 561), (B) Only patients aged under 40 (*n* = 78), (C) patients aged 40 to 49 years (*n* = 237), (D) patients aged 50 to 59 years (*n* = 122), (E) patients aged over 60 (*n* =156). Missing data: CD45: *n* = 30; CD3: *n* = 26; CD8: *n* = 16; FOXP3: *n* = 22; CD163: *n* = 32.

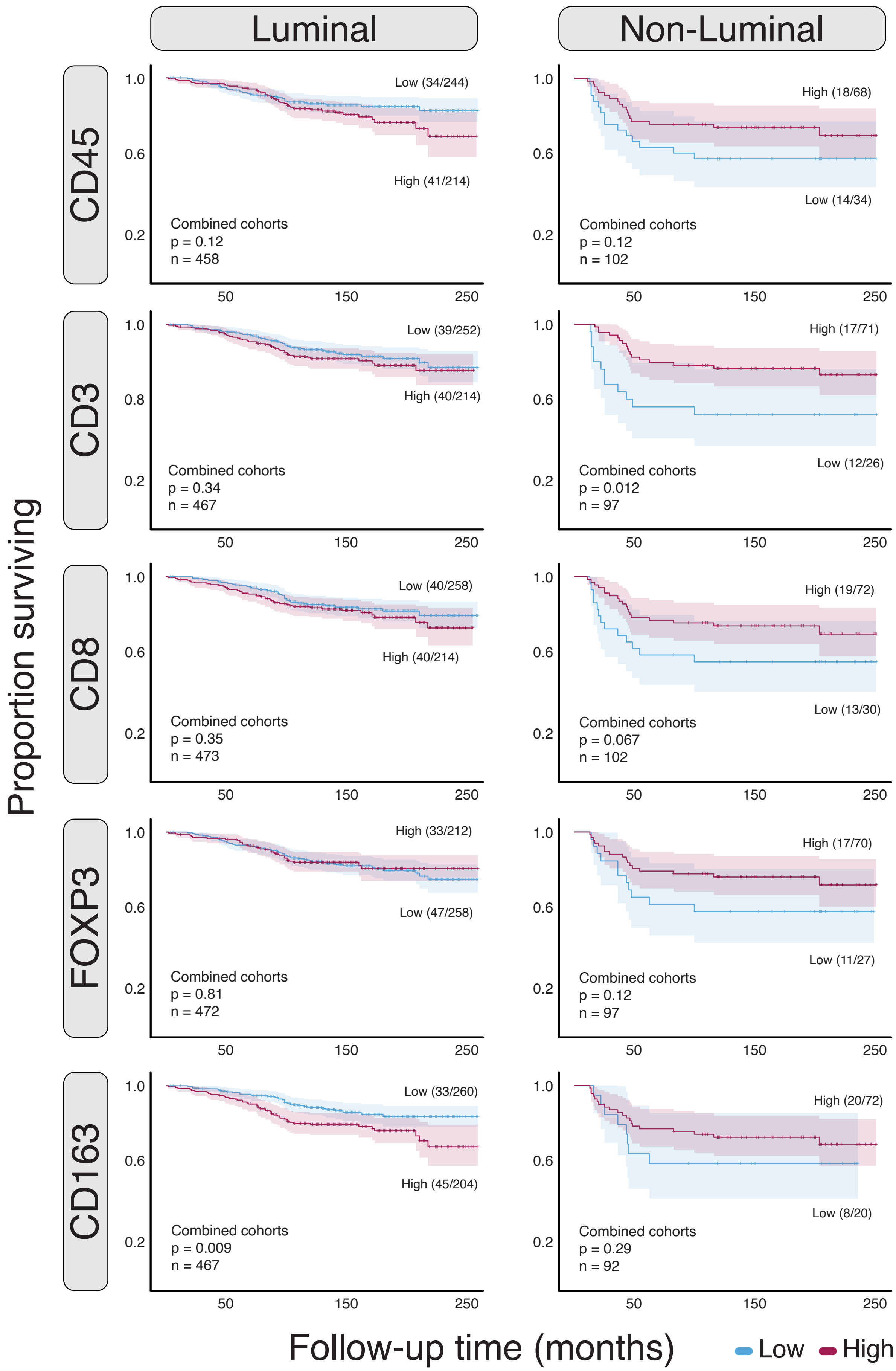

Figure S7. Infiltrating immune cells and patient survival – across subgroups. Breast cancer disease-specific survival according to subtype and immune cell marker staining of CD45; CD3, CD8, FOXP3 and CD163. All markers are dichotomized by the median. Luminal subgroup is defined as Luminal A, Luminal B HER2-negative and Luminal B HER2-positive. Non-Luminal subgroup is defined as HER2-positive (non-luminal) and triple-negative. Kaplan-Meier survival analysis (log-rank test for difference). Missing data: CD45: n = 30; CD3: n = 26; CD8: n = 16; FOXP3: n = 22; CD163: n = 32.

	Observer	Median	Mean	Std Dev	Min	Max
**CD45**	Obs. 1	881	2186	2635	100	11068
	Obs. 2 cor.	811	2370	2947	30	10010
	Obs. 2 raw	292	784	975	10	3312
**CD3**	Obs. 1	799	1391	1522	50	5694
	Obs. 2 cor.	799	1391	1424	50	5858
	Obs. 2 raw	322	576	590	20	2427
**CD8**	Obs. 1	703	703	800	16	3118
	Obs. 2 cor.	706	706	767	21	3155
	Obs. 2 raw	156	342	372	10	1531
**FOXP3**	Obs. 1	101	225	288	0	1338
	Obs. 2 cor.	101	225	267	25	1322
	Obs. 2 raw	41	92	109	10	541
**CD163**	Obs. 1	457	625	509	33	2711
	Obs. 2 cor.	457	601	435	11	2063
	Obs. 2 raw	802	1053	762	20	3614

Table S1. Descriptive information on the inter-observer dataset. Data from the inter-observer cases within the TBC- cohort. “Obs. 1” represents counting from ROCH, the observer of BCY-B cohort. “Obs. 2 cor.” represents counting from YC, the observer of the TC cohort, after adjustments. “Obs. 2 raw” represents counting from YC before adjustments. Counts are standardized to mm^2. *n* = 70, missing n = 11 for all markers.

		CD45 positive staining cells			CD3 positive staining cells			CD8 positive staining cells			FoxP3 positive staining cells			CD163 positive staining cells		
		Low	High		Low	High		Low	High		Low	High		Low	High	
		n (%)	n (%)	P	n (%)	n (%)	P	n (%)	n (%)	P	n (%)	n (%)	P	n (%)	n (%)	P
**Cut-point 8.7%**	**Ki67**															
	Low	71 (49.7)	50 (37.0)	0.034	71 (52.6)	56 (38.4)	0.017	69 (48.3)	62 (41.6)	0.254	105 (60.3)	24 (21.6)	<0.001	71 (57.7)	53 (35.1)	<0.001
	High	72 (50.3)	85 (63.0)		64 (47.4)	90 (61.6)		74 (51.7)	87 (58.4)		69 (39.7)	87 (78.4)		52 (42.3)	98 (64.9)	
	**Molecular subtypes (IHC)**															
	Luminal A	62 (43.7)	39 (28.7)		64 (47.8)	46 (31.1)		62 (43.7)	49 (32.5)		93 (52.8)	19 (17.1)		62 (50.0)	48 (31.4)	
	Luminal B/HER2 neg	41 (28.9)	40 (29.4)		40 (29.9)	42 (28.4)		41 (28.9)	44 (29.1)		46 (26.1)	38 (34.2)		36 (29.0)	46 (30.1)	
	Luminal B/HER2 pos	13 (9.2)	11 (8.1)	0.014	12 (9.0)	11 (7.4)	0.001	14 (9.9)	11 (7.3)	0.052	13 (7.4)	11 (9.9)	<0.001	12 (9.7)	11 (7.2)	<0.001
	HER2 positive	12 (8.5)	14 (10.3)		9 (6.7)	15 (10.1)		11 (7.7)	15 (9.9)		10 (5.7)	13 (11.7)		7 (5.6)	15 (9.8)	
	Triple negative	14 (9.9)	32 (23.5)		9 (6.7)	34 (23.0)		14 (9.9)	32 (21.2)		14 (8.0)	30 (27.0)		7 (5.6)	33 (21.6)	
**Cut-point 10.0%**	**Ki67**															
	Low	75 (52.4)	56 (41.5)	0.067	76 (56.3)	61 (41.8)	0.015	73 (51.0)	68 (45.6)	0.355	110 (63.2)	29 (26.1)	<0.001	75 (61.0)	57 (37.7)	<0.001
	High	68 (47.6)	79 (58.5)		59 (43.7)	85 (58.2)		70 (49.0)	81 (54.4)		64 (36.8)	82 (73.9)		48 (39.0)	94 (62.3)	
	**Molecular subtypes (IHC)**															
	Luminal A	66 (46.5)	42 (30.9)		69 (51.5)	49 (33.1)		66 (46.5)	52 (34.4)		98 (55.7)	22 (19.8)		66 (53.2)	51 (33.3)	
	Luminal B/HER2 neg	37 (26.1)	37 (27.2)		35 (26.1)	39 (26.4)		37 (26.1)	41 (27.2)		41 (23.3)	35 (31.5)		32 (28.5)	43 (28.1)	
	Luminal B/HER2 pos	13 (9.2)	11 (8.1)	0.014	12 (9.0)	11 (7.4)	<0.001	14 (9.9)	11 (7.3)	0.047	13 (7.4)	11 (9.9)	<0.001	12 (9.7)	11 (7.2)	<0.001
	HER2 positive	12 (8.5)	14 (10.3)		9 (6.7)	15 (10.1)		11 (7.7)	15 (9.9)		10 (5.7)	13 (11.7)		7 (5.6)	15 (9.8)	
	Triple negative	14 (9.9)	32 (23.5)		9 (6.7)	34 (23.0)		14 (9.9)	32 (21.2)		14 (8.0)	30 (27.0)		7 (5.6)	33 (21.6)	
**Cut-point 14.0%**	**Ki67**															
	Low	86 (60.1)	64 (47.4)	0.033	86 (63.7)	68 (46.6)	0.004	83 (58.0)	77 (51.7)	0.275	123 (70.7)	35 (31.5)	<0.001	86 (69.9)	64 (42.4)	<0.001
	High	57 (39.9)	71 (52.6)		49 (36.3)	78 (53.4)		60 (42.0)	72 (48.3)		51 (29.3)	76 (68.5)		37 (30.1)	87 (57.6)	
	**Molecular subtypes (IHC)**															
	Luminal A	76 (53.5)	47 (34.6)		78 (58.2)	53 (35.8)		74 (52.1)	59 (39.1)		108 (61.4)	27 (24.3)		74 (59.7)	57 (37.3)	
	Luminal B/HER2 neg	27 (19.0)	32 (23.5)		26 (19.4)	35 (23.6)		29 (20.4)	34 (22.5)		31 (17.6)	30 (27.0)		24 (19.4)	37 (24.2)	
	Luminal B/HER2 pos	13 (9.2)	11 (8.1)	0.006	12 (9.0)	11 (7.4)	<0.001	14 (9.9)	11 (7.3)	0.043	13 (7.4)	11 (9.9)	<0.001	12 (9.7)	11 (7.2)	<0.001
	HER2 positive	12 (8.5)	14 (10.3)		9 (6.7)	15 (10.1)		11 (7.7)	15 (9.9)		10 (5.7)	13 (11.7)		7 (5.6)	15 (9.8)	
	Triple negative	14 (9.9)	32 (23.5)		9 (6.7)	34 (23.0)		14 (9.9)	32 (21.2)		14 (8.0)	30 (27.0)		7 (5.6)	33 (21.6)	

Table S2. **KI67 sensitivity analyses.** Sensitivity analysis of different KI67 cut-points. Data from BCY-B cohort only. *P* -values by Pearson's chi-square test. Missing data: CD45: *n* = 30; CD3: *n* = 26; CD8: *n* = 16; FOXP3: *n* = 22; CD163: *n* = 32; Ki67: *n* = 8; Molecular subgroups: *n* = 5.

	CD45 positive staining cells Median (73)			CD3 positive staining cells Median (56)			CD8 positive staining cells Median (24)			FOXP3 positive staining cells Median (4)			CD163 positive staining cells Median (28)		
	Low	High		Low	High		Low	High		Low	High		Low	High	
	n (%)	n (%)	P	n (%)	n (%)	P	n (%)	n (%)	P	n (%)	n (%)	P	n (%)	n (%)	P
**Age at diagnosis (yrs)**															
<40	30 (41.7)	42 (58.3)	0.25	33 (44.6)	41 (55.4)	0.56	37 (48.1)	40 (51.9)	0.91	39 (52.0)	36 (48.0)	<0.001	34 (49.3)	35 (50.7)	0.098
40-49	116 (54.5)	97 (45.5)		107 (49.8)	108 (50.2)		109 (49.1)	113 (50.9)		141 (64.7)	77 (35.3)		95 (44.4)	119 (55.6)	
50-59	58 (47.5)	66 (52.5)		57 (46.7)	65 (53.3)		63 (51.6)	59 (48.4)		43 (35.2)	79 (64.8)		65 (53.3)	57 (46.7)	
≥60	76 (48.7)	80 (51.3)		84 (53.8)	72 (46.2)		81 (51.9)	75 (48.1)		66 (42.3)	90 (57.7)		89 (57.1)	67 (42.9)	
**Tumor diameter**															
≤2.0 cm	185 (49.6)	188 (50.4)	0.83	184 (49.1)	191 (50.9)	0.65	189 (49.6)	192 (50.4)	0.75	196 (52.1)	180 (47.9)	0.31	204 (54.7)	169 (45.3)	0.009
>2.0 cm	94 (50.5)	92 (49.5)		96 (51.1)	92 (48.9)		98 (51.0)	94 (49.0)		91 (47.6)	100 (52.4)		79 (42.9)	105 (57.1)	
**Nodal status**															
Negative	172 (52.0)	159 (48.0)	0.12	179 (53.4)	156 (46.6)	0.019	184 (54.8)	152 (45.2)	0.010	175 (51.5)	165 (48.5)	0.62	184 (54.9)	151 (45.1)	0.009
Positive	105 (46.5)	121 (53.5)		98 (43.4)	128 (56.6)		103 (43.8)	132 (56.2)		111 (49.3)	114 (50.7)		96 (43.6)	124 (56.4)	
**Histological grade**															
Low (grade 1)	72 (54.5)	60 (45.5)	0.19	84 (60.9)	54 (39.1)	0.003	82 (59.9)	55 (40.1)	0.011	85 (62.5)	51 (37.5)	0.002	90 (66.2)	46 (33.8)	<0.001
High (grade 2 & 3)	200 (48.1)	216 (51.9)		191 (46.0)	224 (54.0)		202 (47.4)	224 (52.6)		198 (47.1)	222 (52.9)		187 (45.6)	223 (54.4)	
**ER**															
Positive	231 (53.6)	200 (46.4)	<0.001	240 (54.5)	200 (45.5)	<0.001	247 (55.4)	199 (44.6)	<0.001	242 (54.4)	203 (45.6)	<0.001	250 (56.9)	189 (43.1)	<0.001
Negative	49 (37.1)	83 (62.9)		41 (32.3)	86 (67.7)		43 (32.8)	88 (67.2)		47 (37.3)	79 (62.7)		33 (27.0)	89 (73.0)	
**PR**															
Positive	196 (53.1)	173 (46.9)	0.027	205 (54.4)	172 (45.6)	0.001	207 (54.2)	175 (45.8)	0.008	211 (55.2)	171 (44.8)	0.002	211 (56.0)	166 (44.0)	<0.001
Negative	84 (43.3)	110 (56.7)		76 (40.0)	114 (60.0)		83 (42.6)	112 (57.4)		78 (41.3)	111 (58.7)		72 (39.1)	112 (60.9)	
**HER2**															
Negative	244 (50.4)	240 (49.6)	0.42	245 (50.0)	245 (50.0)	0.39	253 (50.9)	244 (49.1)	0.32	260 (52.5)	235 (47.5)	0.010	256 (52.6)	231 (47.4)	0.005
Positive	35 (45.5)	42 (54.5)		33 (44.6)	41 (55.4)		35 (44.9)	43 (55.1)		27 (36.5)	47 (63.5)		25 (34.7)	47 (65.3)	
**Ki67**															
Low	152 (55.3)	123 (44.7)	0.011	157 (55.9)	124 (44.1)	0.003	158 (55.4)	127 (44.6)	0.015	178 (62.9)	105 (37.1)	<0.001	173 (62.2)	105 (37.8)	<0.001
High	127 (44.6)	158 (55.4)		122 (43.3)	160 (56.7)		131 (45.3)	158 (54.7)		107 (37.7)	177 (62.3)		107 (38.5)	171 (61.5)	
**Molecular subtypes (IHC)**															
Luminal A	141 (56.4)	109 (43.6)	0.003	149 (57.5)	110 (42.5)	<0.001	150 (57.9)	109 (42.1)	<0.001	163 (62.9)	96 (37.1)	<0.001	161 (62.9)	95 (37.1)	<0.001
Luminal B/HER2 neg	81 (48.8)	85 (47.6)		82 (49.1)	85 (50.9)		85 (50.0)	85 (50.0)		80 (47.3)	89 (52.7)		82 (49.1)	85 (50.9)	
Luminal B/HER2 pos	22 (52.4)	20 (47.6)		21 (51.2)	20 (48.8)		23 (53.5)	20 (46.5)		15 (35.7)	27 (64.3)		17 (41.5)	24 (58.5)	
HER2 positive	13 (37.1)	22 (62.9)		12 (36.4)	21 (63.6)		12 (34.3)	23 (65.7)		12 (37.5)	20 (62.5)		8 (25.8)	23 (74.2)	
Triple negative	21 (31.3)	46 (68.7)		14 (21.9)	50 (78.1)		18 (26.9)	49 (73.1)		15 (23.1)	50 (76.9)		12 (19.7)	49 (80.3)	
**Luminal vs Non-Luminal**															
Luminal	244 (53.3)	214 (46.7)	<0.001	252 (54.0)	215 (46.0)	<0.001	258 (54.7)	214 (45.3)	<0.001	258 (54.9)	212 (45.1)	<0.001	260 (56.0)	204 (44.0)	<0.001
Non-Luminal	34 (33.3)	68 (66.7)		26 (26.8)	71 (73.2)		30 (29.4)	72 (70.6)		27 (27.8)	70 (72.2)		20 (21.7)	72 (78.3)	

Table S3. Clinico-pathologic features and infiltrating immune cells. Evaluation of tumor infiltrating immune cells dichotomized as “low” or “high” staining by median in relation to clinico-pathological features. Patients under 50 years are from the BCY-B cohort, while patients over 50 years are from the TBC cohort. *P*-values by Pearson's chi-square test. Missing data: CD45: *n* = 30; CD3: *n* = 26; CD8: *n* = 16; FOXP3: *n* = 22; CD163: *n* = 32; ER:*n* = 0; PR: *n* = 0; HER2: *n* = 4; lymph node status: *n* = 6; tumor diameter: *n* = 4; tumor grade: *n* = 15; Ki67: *n* = 8; Molecular subtypes: *n* = 5.
